# Epigenome erosion and SOX10 drive neural crest phenotypic mimicry in triple-negative breast cancer

**DOI:** 10.1038/s41523-022-00425-x

**Published:** 2022-05-02

**Authors:** Jodi M. Saunus, Xavier M. De Luca, Korinne Northwood, Ashwini Raghavendra, Alexander Hasson, Amy E. McCart Reed, Malcolm Lim, Samir Lal, A. Cristina Vargas, Jamie R. Kutasovic, Andrew J. Dalley, Mariska Miranda, Emarene Kalaw, Priyakshi Kalita-de Croft, Irma Gresshoff, Fares Al-Ejeh, Julia M. W. Gee, Chris Ormandy, Kum Kum Khanna, Jonathan Beesley, Georgia Chenevix-Trench, Andrew R. Green, Emad A. Rakha, Ian O. Ellis, Dan V. Nicolau, Peter T. Simpson, Sunil R. Lakhani

**Affiliations:** 1 The University of Queensland Faculty of Medicine, UQ Centre for Clinical Research, Herston, QLD Australia; 2 Mater Research Institute-The University of Queensland, Translational Research Institute, Woolloongabba, QLD Australia; 3 School of Mathematical Sciences, Queensland University of Technology, Brisbane, QLD Australia; 4 QIMR Berghofer Medical Research Institute, Brisbane, QLD Australia; 5 Breast Cancer Molecular Pharmacology Unit, School of Pharmacy and Pharmaceutical Sciences, Cardiff University, Cardiff, UK; 6 The Kinghorn Cancer Centre, Garvan Institute of Medical Research and St. Vincent’s Hospital Clinical School, UNSW Sydney, Darlinghurst, NSW Australia; 7 Nottingham Breast Cancer Research Centre, Academic Unit for Translational Medical Sciences, School of Medicine, University of Nottingham Biodiscovery Institute, University Park, Nottingham, UK; 8 Mathematical Institute, University of Oxford, and Molecular Sense Ltd, Oxford, UK; 9 Pathology Queensland, Royal Brisbane Women’s Hospital, Herston, QLD Australia

**Keywords:** Breast cancer, Tumour heterogeneity

## Abstract

Intratumoral heterogeneity is caused by genomic instability and phenotypic plasticity, but how these features co-evolve remains unclear. SOX10 is a neural crest stem cell (NCSC) specifier and candidate mediator of phenotypic plasticity in cancer. We investigated its relevance in breast cancer by immunophenotyping 21 normal breast and 1860 tumour samples. Nuclear SOX10 was detected in normal mammary luminal progenitor cells, the histogenic origin of most TNBCs. In tumours, nuclear SOX10 was almost exclusive to TNBC, and predicted poorer outcome amongst cross-sectional (*p* = 0.0015, hazard ratio 2.02, *n* = 224) and metaplastic (*p* = 0.04, *n* = 66) cases. To understand SOX10’s influence over the transcriptome during the transition from normal to malignant states, we performed a systems-level analysis of co-expression data, de-noising the networks with an eigen-decomposition method. This identified a core module in SOX10’s normal mammary epithelial network that becomes rewired to NCSC genes in TNBC. Crucially, this reprogramming was proportional to genome-wide promoter methylation loss, particularly at lineage-specifying CpG-island shores. We propose that the progressive, genome-wide methylation loss in TNBC simulates more primitive epigenome architecture, making cells vulnerable to SOX10-driven reprogramming. This study demonstrates potential utility for SOX10 as a prognostic biomarker in TNBC and provides new insights about developmental phenotypic mimicry—a major contributor to intratumoral heterogeneity.

## Introduction

Effective management of triple-negative breast cancer (TNBC) remains a significant challenge worldwide. These tumours lack expression of oestrogen and progesterone receptors (ER/PR) and HER2, hence are not indicated for treatment with classical molecular-targeted agents. Chemotherapy remains the most reliable systemic treatment option, producing durable responses in ~60% of patients, while the other ~40% typically present with lung, liver and/or brain metastases within 5 years^1–3^. Second-line chemotherapy can temporarily stabilise metastatic disease but is rarely curative, so these patients endure a heavy treatment burden for no lasting benefit. Efforts to develop alternative treatments have been hampered by molecular and cellular variability between, and within, individual tumours. Intra-tumoural heterogeneity (ITH) directly increases the probability of relapse because it diversifies the substrate for clonal selection^4–7^. It has been proposed that to further improve the prognosis for TNBC patients, we need to develop agents that target the drivers of heterogeneity itself^8^.

TNBCs are characterised by defective DNA repair, mitotic spindle dysfunction, chromosomal aberrations, and a mutation rate around 13 times that of other breast tumours^4,5^. Genomic instability is a key driver of ITH, however only some cases can be explained by the selection of individual driver mutations^9^, and other sources of heterogeneity are coming to light^10–12^. For example, cellular heterogeneity is influenced by the differentiation state of the normal cellular precursor(s)^13^, which in TNBC is thought to be the luminal progenitor (LP) cell^14–17^.

ITH is also driven by phenotypic plasticity—the dynamic reprogramming of cell state in response to extrinsic stimuli^10,11^. Cancer cell state transitions can be de-differentiating (the loss of lineage commitment and acquisition of stem cell features) and/or trans-differentiating (assuming the state of another cell type)^18^. Compared to genomic and histogenic sources of ITH, how tumour cells invoke this capability is poorly understood, and yet potentially more ominous for the patient, as cell state transitions can be induced by treatment via heritable-epigenetic change. In controlled experimental conditions, drug-tolerant TNBC cell states can be averted by epigenome remodelling inhibitors^19–23^, suggesting these agents might reduce rates of relapse if used clinically^8,11^. However, epigenetic therapies have genome-wide effects, so our ability to use them rationally requires a deeper understanding of the epigenome-driven features of treatment-refractory human tumours^8^.

SOX10 is a transcription factor that was recently implicated in phenotypic plasticity in experimental models of TNBC^24^. It is first expressed in embryonic neural crest stem cells (NCSCs), where its self-reinforcing gene regulatory module facilitates multipotency and cell migration, orchestrating the embryo patterning process^25–28^. Once patterning is complete, *SOX10* is silenced in all NCSC descendants except glial and melanocyte progenitors; and is nascently induced in ectoderm-derived epithelial progenitor cells of the salivary, lacrimal, and mammary glands^29–33^. In the mouse, Sox10 is an obligate requirement for mammary gland development. Its expression marks gland repopulating potential in the basal (myoepithelial) compartment, while Sox10+ luminal cells represent the committed progenitor fraction^29^. Functional studies have shown that Sox10 is one of several fate specifiers that regulates the equilibrium between mammary stem cell (MaSC) and LP states^29,32^.

In NCSCs where the genome is unmethylated and accessible, SOX10 facilitates a mesenchymal, migratory state, whereas its function in adult tissues is influenced by the tissue-specific growth factor milieu and lineage-specific DNA methylation. Remarkably, ectopic expression of *SOX10* reprogrammed postnatal fibroblasts with multipotency and migration capabilities equivalent to NCSCs, providing they were also exposed to chromatin unpacking agents and early morphogens (DNA methylation and histone deacetylase inhibitors plus Wnt activation)^34^. This established that with the erasure of lineage-specific epigenetic marks and appropriate extrinsic cues, SOX10 can recreate its ‘default’ regulatory circuit and that this is sufficient to phenocopy NCSCs.

SOX10 expression in human breast cancer is associated with TN, basal-like, metaplastic and neural progenitor-like phenotypes^4,35–39^. In transgenic mouse mammary tumour cells, it promoted invasiveness, expression of mammary stem/progenitor, EMT and NCSC genes and the repression of epithelial differentiation genes^24^. These findings suggest that SOX10 could mediate de-differentiation in TNBC; but the relevance is unclear, particularly given there are no available inhibitors of SOX10 itself. We explored the significance of SOX10 in breast cancer development and progression by immunophenotyping histologically normal breast tissue, and large breast tumour sample cohorts. To understand its contribution to phenotypic plasticity and identify drivers of this capability, we performed systems-level analysis to map SOX10’s regulatory circuit in the broader TNBC transcriptional network.

## Results

### SOX10 is expressed in luminal progenitor cells of the human mammary gland

**Fig. 1:**
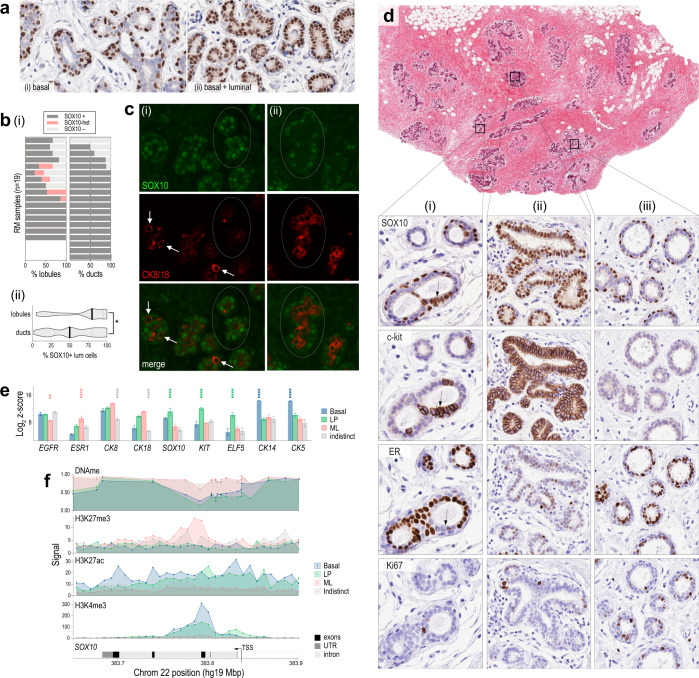
SOX10 is expressed in basal and luminal progenitor cells of the human mammary gland. **a** Representative SOX10 IHC analysis of reduction mammoplasty (RM) samples. Some terminal ducto-lobular units (TDLUs) had exclusive basal compartment expression (i) while others had expression in both basal and luminal compartments (ii). **b** (i) Analysis of SOX10 expression in ducts vs lobules of RM samples from 19 donors (whole sections). (ii) SOX10 expression in lobules was heterogeneous and more likely to occur in the luminal compartment (Mann–Whitney *p* = 0.011; *n* = 102 ducts and 102 lobules; median ± 95% confidence interval shown). **c** Representative immunofluorescent staining of SOX10 and CK8/18. Circled lobules and isolated cells (arrows) exhibited reciprocal expression of SOX10 (green) and CK8/18 (red) in structures with either (i) dual compartment (ii) or basal-restricted SOX10 expression. **d** IHC analysis of SOX10, c-kit, ER and Ki67 in serial RM sections. The three magnified regions represent major SOX10 staining patterns: (i) dual compartment, heterogeneous; (ii) dual compartment, homogeneous; and (iii) basal-restricted. Luminal SOX10 expression was directly associated with c-kit and inversely associated with ER, with no obvious relationship to Ki67 (e.g., cell cluster indicated with an arrow). **e** SOX10 mRNA levels in FACS-sorted human mammary epithelial cell (hMEC) subtypes^15^. Differentiation markers were analysed for comparison: basal markers CK14 and CK5; luminal progenitor (LP) markers KIT and ELF5; and markers enriched in mature luminal (ML) cells: CK18 and ESR1 (isolates with significantly different marker levels according to paired ANOVA tests are indicated and colour-coded: ****p < 0.00001; ***p < 0.0001; **p < 0.001). Data shown were means ± standard error of the mean from three donors. **f** Average methylation beta-values of SOX10 probes in FACS-sorted hMEC samples (DNAme), aligned with histone modification signals in a published ChIP-seq dataset^42^: H3K4me3, H3K27ac (activating) and H3K27me3 (repressive). Data were represented to scale on human chromosome 22. TSS transcription start site, UTR untranslated region. Indistinct = negative for CD45 (hematopoietic cells), CD31 (endothelia), CD140b (fibroblasts), EpCAM and CD49f (epithelia).

Functional studies have shown that SOX10 marks stem and luminal progenitor (LP) cells of the mouse mammary gland^29,32^, but its expression pattern in the human breast has not been established. Therefore, we performed immunohistochemical (IHC) analysis of 19 histologically normal reduction mammoplasty (RM) samples using a validated antibody (Supplementary Fig. 1a and Supplementary Table 1). SOX10 was detected in nuclei of ductal and lobular epithelia, with individual terminal ducto-lobular units (TDLUs) exhibiting either basal-restricted or combined baso-luminal expression (Fig. 1a). Compared to ducts, lobules were more likely to exhibit luminal compartment expression of SOX10 (Fig. 1b), consistent with a role in lobulogenesis. Indeed, TDLUs with basal-restricted SOX10 expressed high levels of luminal cytokeratins (CK)8/18, while TDLUs with dual-compartment SOX10 had low CK8/18. This was evident even in neighbouring structures of the same specimen (Fig. 1c and Supplementary Fig. 1b).

IHC analysis of serial sections showed SOX10+ luminal cells lacked ER and were positive for the LP marker c-Kit, with no obvious relationship to proliferation marker Ki67 (Fig. 1d). We also analysed *SOX10* mRNA in a published dataset from FACS-sorted human mammary epithelial cells (hMECs)^15^. *SOX10* levels were similar to established LP markers *ELF5* and *KIT*: highest in EpCAM + /CD49f + LP cells, moderate in the EpCAM-/CD49f + basal compartment (myoepithelia and mammary stem cells (MaSCs)) and low in EpCAM + /CD49f- mature luminal (ML) cells (Fig. 1e).

SOX10 is epigenetically regulated in mouse mammary gland^40,41^, so we investigated this in human tissue. We isolated hMECs from two fresh RM samples using FACS with antibodies against CD49f and EpCAM, then performed high-density DNA methylation array profiling. *SOX10* was hypomethylated in LP and basal samples (*p* < 1.0E^−06^; Fig. 1f). Consistently, analysis of hMEC chromatin immunoprecipitation sequencing (ChIP-seq) data from six independent RM samples^42^ showed the *SOX10* locus is enriched with activating (H3K4me3, H3K27ac) and depleted of repressive H3K27me3 marks in LP and basal samples (Fig. 1f).

### SOX10 is associated with poor clinical outcomes in TNBC

**Fig. 2:**
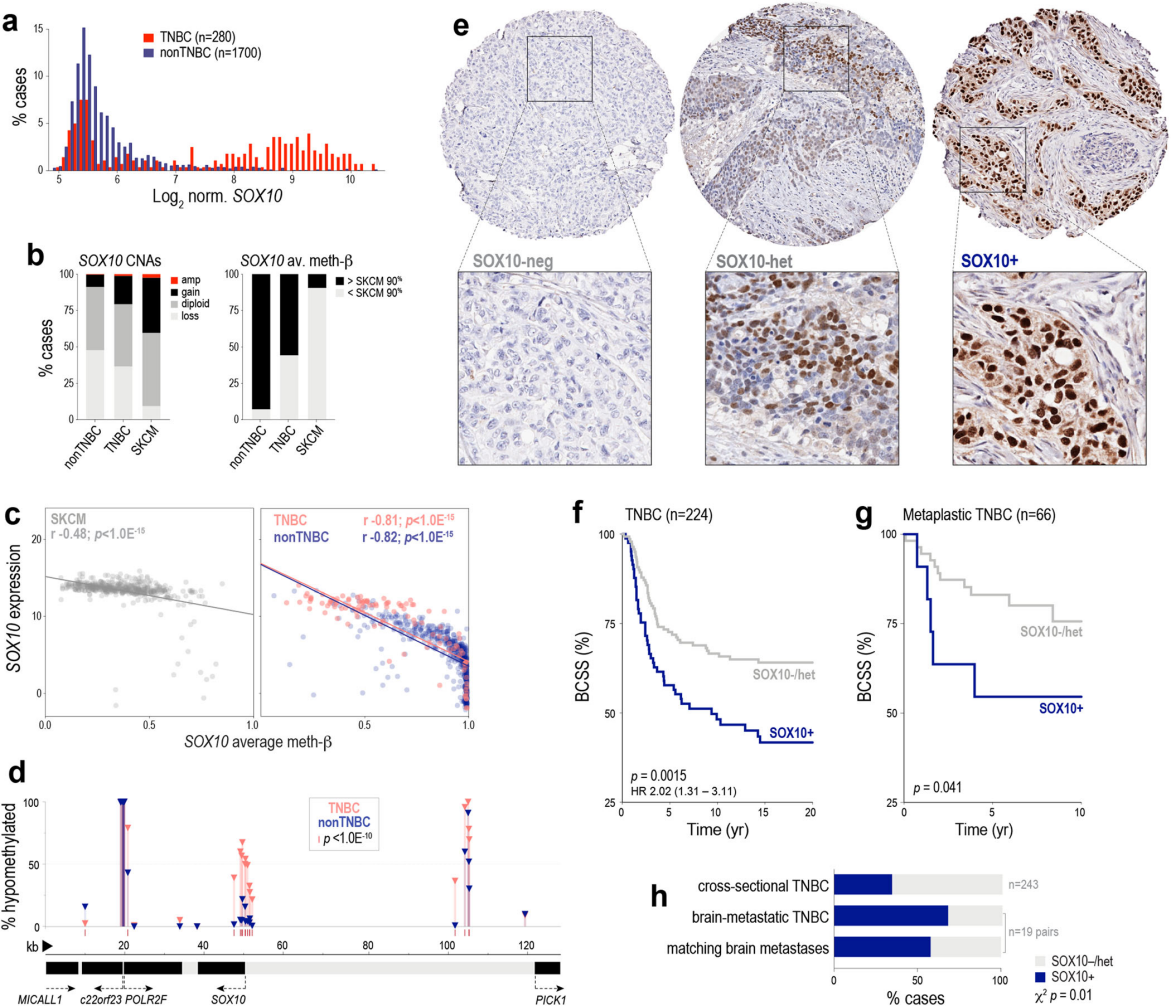
Expression of SOX10 in human breast cancer. **a** Bimodal expression of *SOX10* in TNBC compared to other breast cancers (nonTNBC) in the METABRIC cohort. **b** Frequency of copy-number alterations (CNAs) and DNA hypomethylation affecting *SOX10* in TNBC and nonTNBC compared to the archetypal SOX10 + malignancy, melanoma (SKCM; TCGA datasets). **c** Correlation between *SOX10* methylation and expression (normalised RNAseq counts) in SKCM, TNBC and nonTNBC (Spearman correlation coefficients (r) and *p* values are shown; derived from TCGA data). **d** Proportions of TNBC and nonTNBC cases with hypomethylation at each probe across the *SOX10* locus (as defined in (**b**)). **e** Representative IHC showing SOX10-neg, heterogeneous and nuclear-positive (+) TNBCs. Tumours with absent or very weak nuclear staining in ≥50% of tumour cells were classified as SOX10-negative, while those with any one of replicate TMA cores exhibiting moderate-strong nuclear staining in <50% OR weak-moderate nuclear staining in ≥50% of tumour cells were classified as heterogeneous (see also Supplementary Fig. 2h). Survival curves of heterogeneous and negative categories overlapped (Supplementary Fig. 2j) and hence are grouped together here. **f** Kaplan–Meier analysis of the relationship between SOX10 nuclear positivity and breast cancer-specific survival (BCSS) in cross-sectional TNBCs. Log-rank test *p* value and hazard ratio (HR) are shown (95% confidence interval). **g** Kaplan–Meier analysis of the relationship between SOX10 nuclear positivity and BCSS in TNBCs classified as metaplastic breast cancers. Gehan–Breslow–Wilcoxon test *p* value shown. **h** SOX10 expression in brain-metastatic TNBC and matching brain metastases (BrM), compared to the frequency in cross-sectional TNBCs (Chi-square *p* value shown).

Analysis of TCGA, METABRIC and ICGC breast tumour datasets^43–45^ showed *SOX10* mRNA is expressed almost exclusively in TNBC, with a bimodal distribution suggesting distinct SOX10 positive and negative (+/−) subgroups (Fig. 2a and Supplementary Fig. 2a). Consistent with other data^39^, *SOX10* mRNA is highest amongst TNBCs classified as ‘basal-like, immune-suppressed’ (BLIS), though we noted that expression was heterogeneous amongst TNBC subtypes classified by gene expression profile (e.g. 23% of ‘basal-like, immune-activated’ (BLIA) TNBCs also had *SOX10* levels in the top quartile; Supplementary Fig. 2b). In terms of genomic drivers of SOX10 expression in breast cancer, copy-number (CN) amplification or gain at the *SOX10* locus was evident in ~20% of TNBCs (Fig. 2b) and was associated with higher mRNA levels in both METABRIC and TCGA datasets (Fisher’s Exact *p* ≤ 0.001). Analysis of TCGA HM450k methylation array data indicated that *SOX10* is frequently hypomethylated in TNBC (Fig. 2b) and that this correlates strongly with expression (Fig. 2c and Figs. S2c, d), but does not extend to adjacent genes on chromosome 22 (Fig. 2d). Hence, like normal basal and luminal progenitor cells, gene-specific hypomethylation also underpins *SOX10* expression in a subset of TNBCs, and in some cases, this appears to be reinforced by clonally selected CN gains.

Analysing published cell line gene expression and methylation array datasets^46,47^ and our cell line bank^48,49^, we found that in contrast to tumours, TNBC cell lines express very low to undetectable levels of *SOX10*, and the *SOX10* gene is hypermethylated (Fig. S2e, f). shRNA-mediated depletion of *SOX10* in one of the few positive lines (HCC1569) resulted in 100% cell death within a few passages (Supplementary Fig. 2g).

Next, we performed IHC studies to investigate the prognostic significance of SOX10 expression at the protein level. Surveying a large, cross-sectional cohort of invasive primary breast tumours from Australia and the UK (*n* = 1330), we detected SOX10 almost exclusively in tumour cell nuclei of TN cases (Fig. 2e; see Supplementary Table 2 for cohort characteristics). Approximately 38% of TNBCs were classified as SOX10+, and another 11.5% exhibited heterogeneous staining (see Fig. 2e and Supplementary Fig. 2h for scoring thresholds). SOX10 positivity was associated with histologic features typical of this group, such as high grade, metaplastic and medullary morphology, pushing margins and a larger size at diagnosis (Supplementary Table 2). Similar, though statistically weaker trends were found between these variables and heterogeneous SOX10 staining (Supplementary Fig. 2i).

Rather than a simple correlate of the TN phenotype, SOX10 positivity stratified TNBC-specific survival in both univariate (Fig. 2f and Supplementary Fig. 2j) and multivariate regression analyses, with a prognostic value greater than clinicopathologic indicators used in current clinical practice: tumour size, grade, and the density of tumour-infiltrating lymphocytes (TILs) (hazard ratio 1.8-2.5; *p* = 0.02–0.002; Supplementary Table 2). Increased propensity for brain metastasis is one of the factors underlying premature death in TNBC, so we also analysed patient-matched pairs of primary TNBCs and brain metastases (*n* = 19 pairs). Compared to cross-sectional TNBCs, SOX10 was over-represented in brain-metastatic cases, with SOX10 status concordant in ~90% of matching brain tumours (Fig. 2h). Consistent with previous reports^37,50^, we also detected nuclear SOX10 in an independent cohort of metaplastic breast cancers (MBC; Asia-Pacific Metaplastic Breast Cancer consortium^51^). Compared to cross-sectional cases, SOX10 staining was more heterogeneous in MBCs, and was not associated with TN status (Supplementary Fig. 2k); but was prognostic amongst MBCs with a TN phenotype (Fig. 2g).

Considering all our IHC study findings, we concluded that strong nuclear expression of SOX10 is associated with TNBC progression.

### SOX10’s TNBC regulatory module confers transcriptomic similarity to NCSCs

To investigate the basis of SOX10’s association with poor patient outcomes, we compared the expression profiles of TNBCs expressing high versus low levels of *SOX10* mRNA and found that *SOX10*^*high*^ tumours were significantly enriched with the expression of mesenchymal, neural, and glial development genes (Supplementary Fig. 3 and Tables S3, S4).

We then mapped *SOX10’s* regulatory neighbourhood within the breast cancer transcriptome using weighted gene co-expression network analysis (WGCNA). This approach quantifies co-variation in gene expression across a biological sample set to identify genes with highly coordinated regulation, which is indicative of functional relatedness^52,53^. We built a network from TCGA breast cancer RNAseq data (*n* = 919 cases) and validated it with datasets from METABRIC (*n* = 1278, expression array) and ICGC (*n* = 342, RNAseq). In this model, all genes expressed above a background threshold are connected (12,588 genes, 12,588^2^ connections). The connection between each gene pair is based on a weighted correlation coefficient, and unsupervised clustering can reveal groups of genes with a high probability of co-functionality (modular transcription programmes). The module eigengene (ME) is a centroid calculated for each module in each sample that represents both module expression and net connection strength.

**Fig. 3:**
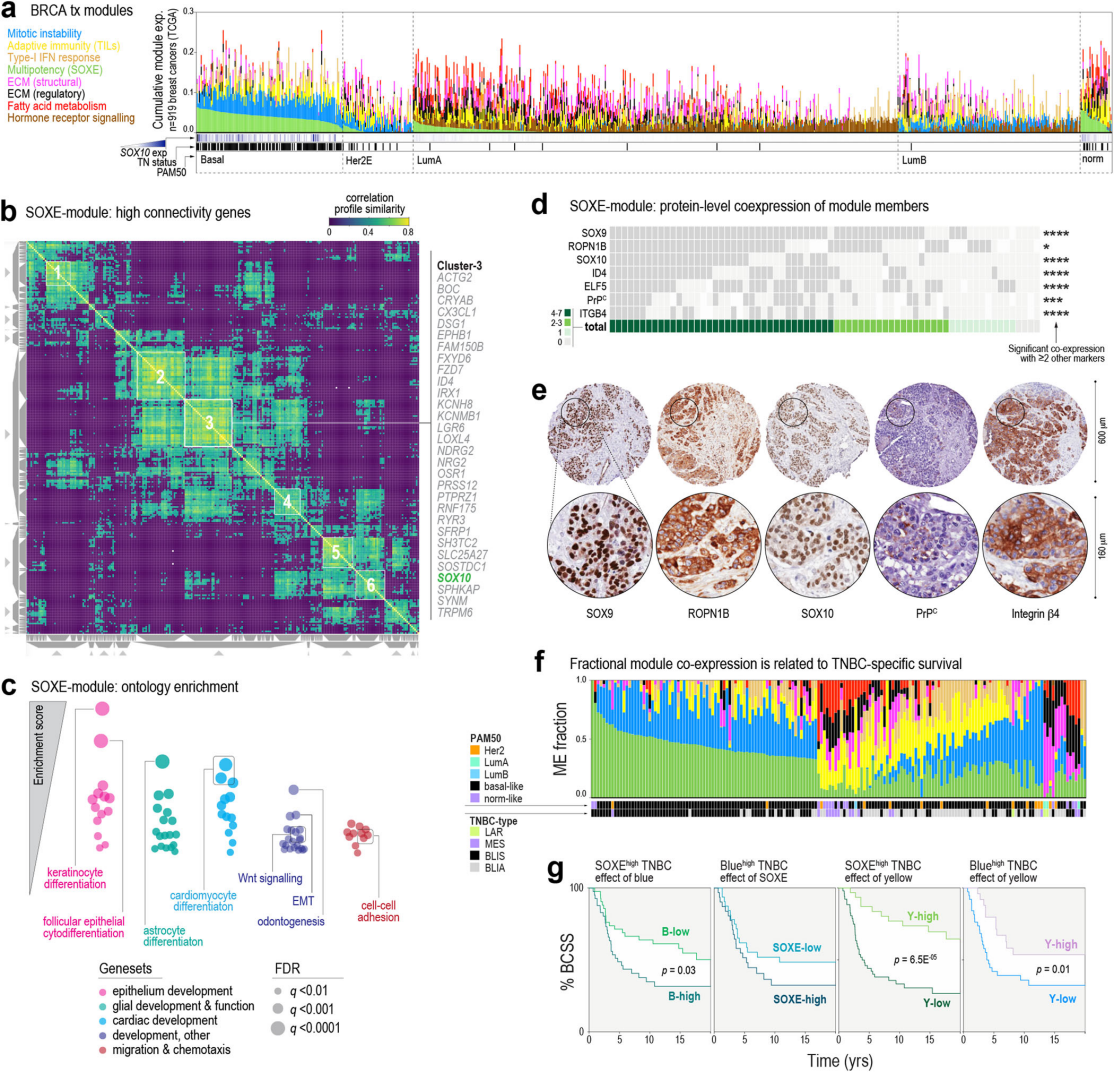
SOX10’s regulatory network is associated with multipotency, cell migration and poor prognosis in TNBC. **a** Relative expression of eight predominant transcription modules in human breast tumours, according to the PAM50 subtype (TCGA dataset). **b** SOXE-module co-expression profile similarity matrix, clustered to highlight genes with very highly coordinated expression. The similarity is based on cosine distance and has a maximum value of 1. SOX10 mapped to one of six module sub-clusters, the members of which are shown to the right of the matrix. See also Supplementary Fig. 4a, b. **c** Summary of results from unsupervised gene set enrichment analysis of the breast cancer transcriptome after ordering transcripts according to their correlations with SOXE-module expression (denoted by the ME value, TCGA dataset). **d** Tile plot showing overlapping expression of SOXE-module representatives. For each protein, significant co-expression with ≥2 other module members is indicated by a Fisher’s exact test result (*p < 0.05; ***p < 0.001; ****p < 0.0001). Refer to Supplementary Table 1 for scoring criteria. **e** IHC staining of representative SOXE-module nodes in serial sections from the same tumour. **f** Proportional expression of all eight modules (coloured as for (**a**)) in TNBCs annotated with PAM50 and TNBC subtypes (METABRIC dataset; LAR luminal androgen receptor-like, MES mesenchymal, BLIS basal-like immune-suppressed, BLIA basal-like immune-activated^39^). **g** Kaplan–Meier analysis of METABRIC TNBCs expressing different proportions of the three predominant TNBC modules. BCSS breast cancer-specific survival. ME fraction thresholds for classifying cases as high or low were 0.33 for SOXE/blue and 0.1 for yellow.

WGCNA partitioned ~20% of expressed genes into eight consensus modules that align with established hallmarks of breast cancer; for example, an ER/FOXA1-driven module expressed in luminal tumours, and a mitotic instability module in basal-like and luminal-B tumours (Table 1, Fig. 3a, Tables S5–S8 and Supp File 2). The remaining ~80% of genes were not linked to any one module. *SOX10* was identified as one of the most interconnected genes in the ‘green’ module, which has a hierarchical structure (Fig. S4a, b) and is predominantly expressed in high-grade TNBCs (Supplementary Fig. 4c). In this module, SOX10’s co-expression profile was highly similar to genes implicated in Wnt signalling, neuroglial differentiation and embryo patterning (Fig. 3b). We named it the SOXE-module and ascribed ‘multipotency’ as its primary ontology, as the member gene list is enriched with developmental phenotypes, and includes all three SOXE family members (*SOX8/9/10*) and embryonic stem cell genes (*LMO4*, *POU5F1*) (Fig. 3c and Supplementary Table 9).

**Table 1:** Key features of eight predominant gene co-expression modules extracted by WGCNA.

Modules		Major functional ontologies^a^	Signalling pathways^a^/intrinsic activators^b^	Size (no. genes)	Top ten hub genes (Highest kWithin; see Supplementary Table 5)
Tumour-centric	Blue	Mitotic instability	FOXM1, MYBL2	1239	TPX2, BUB1, CEP55, HJURP, NCAPH, KIF4A, KIF2C, CCNB2, NCAPG, FOXM1
	Green	Multipotency (SOXE)	Wnt signalling	487	ROPN1, SFRP1, FOXC1, RGMA, GABRP, CHST3, MAML2, APCN, ROPN1B, SOX10
	Brown	Primary cilium	ER, FOXA1	1008	FOXA1, MLPH, ESR1, AGR3, XBP1, THSD4, GATA3, CA12, PRR15, ZMYND10
Tumour-stromal	Magenta	ECM-1 (structural)	FBN1, RUNX2	186	COL5A2, COL1A2, COL3A1, COL5A1, COL6A3, FAP, THBS2, COL1A1, LUM, VCAN
	Black	ECM2 (regulatory)	–	207	OLFML1, RECK, FSTL1, DCN, MSRB3, ECM2, CCDC80, TCF4, ZEB1, GLT8D2
	Red	Fatty acid metabolism	PPARγ	274	DIA1R, PDE2A, LHFP, LDB2, ARHGEF15, S1PR1, SDPR, EBF1, CD34, ERG
	Tan	Type-I IFN response	STAT1, IRF9	33	IFIT3, OAS2, CMPK2, IFI44L, IFI44, IFIT1, MX1, OASL, IFIT2, RSAD2
Stromal	Yellow	Adaptive immunity (TILs)	CD40L, CD40, IFNγ, IRF1	712	SASH3, IL2RG, CD53, PTPN7, CD48, CD2, CD3E, ARHGAP9, CD5, CD3D, SIT1, SH2D1A

*ECM* extracellular matrix.

^a^Gene set enrichment analysis (GSEA) of all BRCA genes ranked according to module eigengene correlation (Supplementary Table 9).

^b^Ingenuity pathways analysis upstream regulator prediction (*p* ≤ 1.0E-07) based on kWithin values for module genes.

IHC analysis of six other module members confirmed that their co-expression in TNBC holds true at the protein level (Fig. 3d), with staining often observed in the same cells within individual tumour-rich tissue cores (Fig. 3e). Consistent with the defining features of TNBCs—de-differentiation, genomic instability, high mitotic index and the presence of TILs—TNBCs express variable proportions of primarily three modules: green (SOXE), blue (mitotic instability) and yellow (TILs) (Fig. 3f). Kaplan–Meier analysis showed that cases expressing high levels of both SOXE and mitotic instability modules had shorter survival compared to those with predominant expression of one or the other, while co-expression of the yellow module was associated with better prognosis, consistent with the protective effect of TILs in TNBC^54^ (Fig. 3g and Supplementary Fig. 4d).

### The SOXE-module represents the shift from a luminal progenitor to an NCSC-like state

**Fig. 4:**
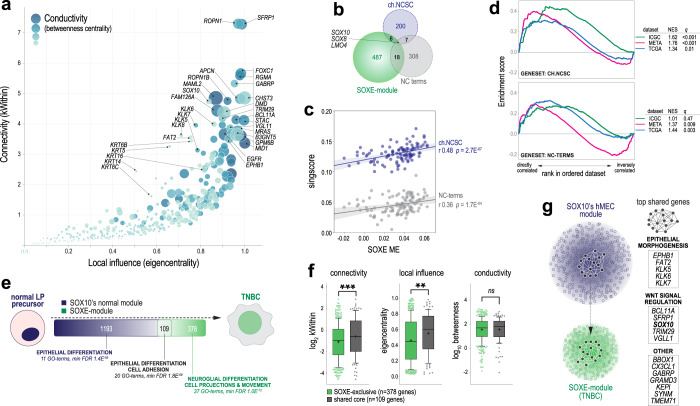
The SOXE-module drives the transition from normal mammary epithelial stem/progenitor to NCSC-like phenotypic states. **a** Influence of SOXE-module genes over network architecture and information flow. kWithin: intramodular ‘connectivity’ based on weighted correlations with all other module genes; Eigencentrality: considers the connectivity of each node’s nearest neighbours as an indicator of ‘local influence’; Betweenness centrality: ‘conductivity’ based on each node’s position along the shortest paths between other nodes (genes with high betweenness are information conduits). Key hub genes are indicated (see Supplementary Table 10 for the full dataset). **b** Chick (ch.)NCSC and neural crest (NC) terms genesets are largely independent of each other and from the SOXE-module. **c** Correlations between SOXE-ME values and NCSC genesets (singscore values) in TNBC (*n* = 106 TCGA cases with tumour cellularity ≥0.6). Correlation coefficients (r) and *p* values are shown. **d** GSEA using three TNBC gene expression datasets (ICGC, METABRIC, TCGA). Normalised enrichment scores (NES) and corrected *p* values (q) shown. **e** Overlap between members of the SOXE-module and SOX10’s normal breast module (from de novo module identification on *n* = 97 TCGA normal breast samples; Supplementary Table 12). Generic ontology enrichment results are summarised (full GO term lists in Supplementary Table 13). **f** Comparison of network structure and information flow metrics (as for (**a**)) between shared and SOXE-module-exclusive genes. Groups were compared using Mann–Whitney tests (**p = 2.4E-03; ***p = 5.6E-04). Boxes show the 10–90th percentiles and median, with whiskers extending to the minimum and maximum values. Mean is indicated with ‘+’. **g** Model depicting the mammary epithelial progenitor gene regulatory network core being sustained through transformation and rewired as the SOXE-module in TNBC. Shared hub genes are listed.

Ontology analysis showed that the SOXE-module includes genes typically expressed in differentiating glia, cardiomyocytes, and odontoblasts, which all descend from NCSCs. In fact, developmental genes comprised a large proportion of SOXE-module hubs (genes with the highest network connectivity and centrality values; Fig. 4a and Supplementary Table 10), hence representing points of maximal module vulnerability. These include cell-fate regulators *ELF5*, *FOXC1* and *SOX10*; Wnt/β-catenin signalling genes *SFRP1*, *MAML2* and *TRIM29*; and embryonic cell migration and neuronal development genes *RGMA*, *ROPN1*, *ROPN1B, MID1* and *APCN*.

To directly investigate if the SOXE-module is associated with NCSC phenotypic mimicry, as has been reported for Sox10 in mouse mammary tumour cells^24^, we performed expression and enrichment analyses using two independent genesets: (1) 308 genes represented in at least two of the 78 terms matching ‘neural crest’ in the gene ontology database (‘NC terms’); and (2) transcripts specific to migratory, Sox10+ NCSCs in chick embryos (‘ch.NCSC’; *n* = 200 genes)^55^, representing Sox10’s most primitive transcription programme (Supplementary Table 11). Except for *SOX10*, *SOX8* and *LMO4*, there is minimal overlap between the SOXE-module and these genesets (Fig. 4b), but their expression is strongly correlated (Fig. 4c). This was confirmed by geneset enrichment analysis (GSEA; Fig. 4d). Hence, the SOXE-module confers transcriptomic similarity to NCSCs.

Since several SOXE-module genes (e.g. *SOX10*, *SOX9*, *LGR6* and *ELF5*) are key regulators of normal hMEC states^56^, we hypothesised that the SOXE-module might evolve from the deregulation of a lineage differentiation programme expressed in TNBC’s normal cellular precursors. Module preservation analysis using RNAseq data from TCGA normal breast samples indicated that the SOXE-module does not exist as an interconnected unit in the normal breast transcriptome (Supplementary Fig. 4e). But after performing *de novo* WGCNA module identification on this dataset (Supplementary Table 12), we found that *SOX10’s* normal breast module overlaps with the TNBC-specific SOXE-module significantly more than expected by chance (Fig. 4e; 109 shared genes, Chi-square *p* = 2.8E^−26^).

Both ‘normal-exclusive’ and ‘shared’ genes were enriched with epithelial differentiation ontologies, with cell adhesion distinctly over-represented in the shared set (Fig. 4e and Supplementary Table 13). According to network influence metrics, the shared genes were significantly more important to the SOXE-module than SOXE-exclusive genes (Fig. 4f and Supplementary Fig. 4f). This suggests that while SOXE-exclusive genes are primarily responsible for conferring NCSC-like attributes, genes ‘inherited’ from TNBC’s normal precursors are comparatively more important to the SOXE-module’s regulatory structure. Together, these data suggest that SOXE-module and its associated NCSC-like phenotype arise because a core set of epithelial differentiation and adhesion genes becomes rewired during TNBC development (Fig. 4g).

### Genomic and epigenomic determinants of the NCSC-like transcriptional shift in TNBC

**Fig. 5:**
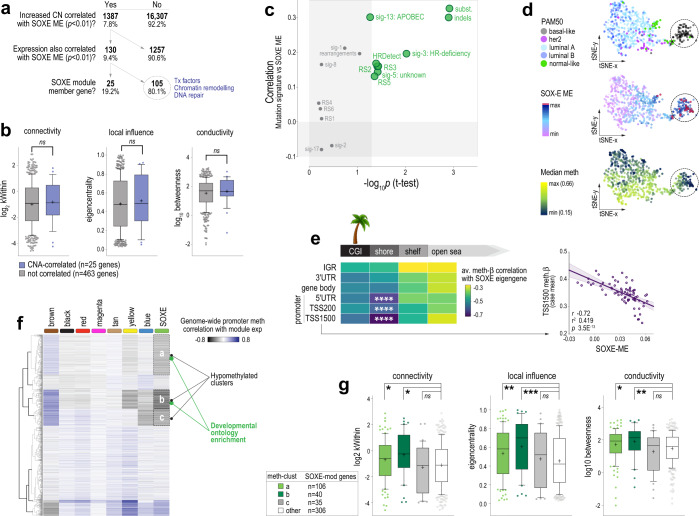
The SOXE-module is driven by the erosion of lineage-specific epigenetic marks. **a** Decision tree for identifying candidate copy-number alteration (CNA) drivers of the SOXE-module. Of 17,694 genes with case-matched GISTIC, RNAseq and WGCNA data, CN, and expression of 130 correlated with the SOXE-module in TNBC, including 25 SOXE-module nodes. **b** Network influence metrics for SOXE-module nodes coloured according to candidate CN driver status (intramodular connectivity (kWithin), local influence (Eigencentrality) and conductivity (betweenness centrality) defined in Fig. 4a). Boxes show the 10–90th percentiles and median, with whiskers extending to the minimum and maximum values. Mean is indicated with ‘+’. No significant differences by ordinary ANOVA test. **c** Relationship between SOXE-module levels and mutation signatures in ICGC TNBCs (COSMIC v2 SigProfiler and HRDetect on *n* = 74 ICGC TNBCs)^45^. Associations are depicted according to the correlation between SOXE-ME values and signature event count (y-axis); and by the significance of average SOXE-ME differences between ICGC TNBCs with low (quartile-1) vs higher (quartile 2–4) signature burden. **d** t-Distributed stochastic neighbour embedding (t-SNE) visualisation of genome methylation profile similarities amongst cases in the BRCA-TCGA 450k methylation array dataset. Panels are coloured according to PAM50 intrinsic subtype, SOXE-ME values or global median methylation-b values. Circled cases are epigenetically divergent, basal-like TNBCs that express high levels of the SOXE-module and have eroded methylomes. **e** Correlation analysis summary showing relationships between SOXE-ME values and region-specific methylation (*n* = 75 TCGA TNBCs, tumour cellularity ≥0.6; *n* = 215,323 probes after quality filtering); ****p < 1.0E-07. CGI CpG island, IGR intergenic region, TSS transcription start site, UTR untranslated region. Solo-WCpGW: consensus sequence for late-replicating loci demethylated via replicative senescence. **f** Unsupervised clustering of the BRCA-TCGA 450k methylation dataset according to ME correlation. Data shown were minimum correlation coefficients of ME values versus gene-averaged methylation-b data from promoter region probes (TSS1500, TSS200 and 5′UTR). Of three clusters inversely correlated with SOXE-module expression, two (**a**, **b**) were enriched with developmental ontologies (Supplementary Table 14). **g** Network influence metrics for SOXE-module genes in the hypomethylated clusters versus other SOXE-module genes, as for (**b**). Ordinary ANOVA *p* values: *p < 0.05; **p < 0.01; ***p < 0.001; ns not significant.

To address the central question of what drives this transcriptomic shift, we analysed case-matched gene copy-number (CN), RNAseq and WGCNA data (TCGA cases). Candidate module drivers were defined as those for which both CN and expression correlated significantly with SOXE-ME values. About 182 genes met these criteria (130 gains and 52 losses), of which 140 (77%) are part of large chromosomal alterations: 6p21-22 (gained/amplified in 56.7% of TNBC cases), 8q22-24 (gained/amplified in 78.7%), 9q34 (lost in 59.6%) (Supplementary Fig. 5a). SOXE-module genes were over-represented amongst the positively correlated genes (25/130 (19.2%) and had increased CN and expression in SOXE^high^ TNBC; ChiSq *p* = 9.7E^−31^; Fig. 5a). However, network influence metrics for these 25 were no higher than other module genes (Fig. 5b). Hence, the SOXE-module may be augmented by increased CN of some of its component genes, but this seemed unlikely to be an early or dominant driver of module evolution.

Next, we investigated whether mutational processes that shape the breast cancer genome could be involved. To this end, we utilised case-matched mutational signature and WGCNA data for the ICGC cohort ^45,57^. There were direct relationships between the SOXE-module and overall mutation burden (substitutions and small insertion-deletion (indels)), as well as specific signatures of genome instability (rearrangement sigs (RS)3 and RS5), homologous recombination (HR)-directed repair of double-strand DNA breaks (DSBs) and genome editing (sig-3: HR deficiency; HRDetect; sig13: APOBEC; Fig. 5c).

APOBEC activity and DSB repair are both indirectly demethylating. For example, 5-methyl cytosine (5mC) loss occurs because of APOBEC-mediated genome editing and/or during the repair of edited bases, and DSB repair has been causally linked to the progressive loss of 5mC during cellular ageing^58,59^. Therefore, we hypothesised that the evolution of the SOXE-module in TNBC may be related to epigenetic dysregulation. Consistent with this idea, the 105 CN-driven SOXE-module correlates (i.e., those not part of the SOXE-module itself; Fig. 5a) were enriched with a transcription factor, chromatin remodelling and DNA repair genes (Fisher’s Exact *p* < 0.001). Furthermore, visualising SOXE-module strength relative to the overall methylome profile using t-SNE showed that SOXE-ME values were highest in the most epigenetically divergent tumours (Fig. 5d).

To investigate this further, we then correlated SOXE-ME values with probe-level methylation data directly, in the following regional categories: CpG islands (CGIs), CGI shores, shelves or open sea regions at transcription start site (TSS) regions, untranslated regions (UTRs), gene bodies or intergenic regions (IGRs). We also quantified methylation at ‘solo-WCpGW’ sites at late-replicating, heterochromatic loci, which act as a biomarker of replicative senescence^60^ and are hypomethylated in breast tumours compared to hMECs (Supplementary Fig. 5b). There was no relationship with solo-WCpGW sites (Supplementary Fig. 5c), but there was a striking inverse correlation between SOXE-ME values and genome-wide promoter methylation; particularly at CGI shores, the substrate for lineage-specific methylation in adult tissues (Fig. 5e and Supplementary Fig. 5c). These data indicate that SOXE-module expression and connectivity are directly proportional to promoter demethylation in TNBC (Fig. 5e). There was no such relationship with any other module in TNBC (Supplementary Fig. 5d).

Having established that SOXE-module levels correspond with loss of tissue-specific 5mC marks, we then built a correlation matrix from ME and genome-wide promoter methylation data (TCGA) and performed unsupervised clustering to look for evidence of epigenetic control. The SOXE-module had a distinct promoter methylation signature—three clusters of genes that are hypomethylated when SOXE-module strength is highest, of which two were enriched with developmental ontologies (Fig. 5f and Supplementary Table 14). Only 10% of these correspond to SOXE-module genes, but this 10% is enriched with hub genes (Fig. 5g), suggesting a higher level of epigenetic control over module structure and information flow. We then used GSEA to test the enrichment of the SOXE-associated promoter methylome with NCSC genesets. Like the transcriptome (Fig. 4d), the methylation landscape associated with the SOXE-module was also enriched with NCSC genes (NC terms: normalised enrichment score (NES) −1.5; *q* = 6.0E^−03^; Ch.NCSC: NES −1.3; *q* = 3.6E^−02^).

Finally, we investigated direct demethylation processes as potential enablers of SOXE-module formation by cross-referencing SOXE-ME values from our three WGCNA datasets (TCGA, ICGC, METABRIC) against the expression of demethylases in the EpiFactors database^61^. There were direct associations with APOBEC3A/3B cytosine deaminases and *TET1* (Supplementary Fig. 5e). TET dioxygenase enzymes catalyse the first step of 5mC demethylation and are involved in processes requiring cell states to be reset or adjusted, such as methylome erasure in preimplantation embryos, and epigenetic plasticity in brain regions that facilitate learning and memory. *TET1* is a maintenance demethylase that prevents methylation from spreading from silenced loci, particularly at CGI shores^62,63^. It has been causally implicated in TNBC metastasis^64^ and our findings suggest this may be at least partly due to reinforcement of the SOXE-module.

**Fig. 6:**
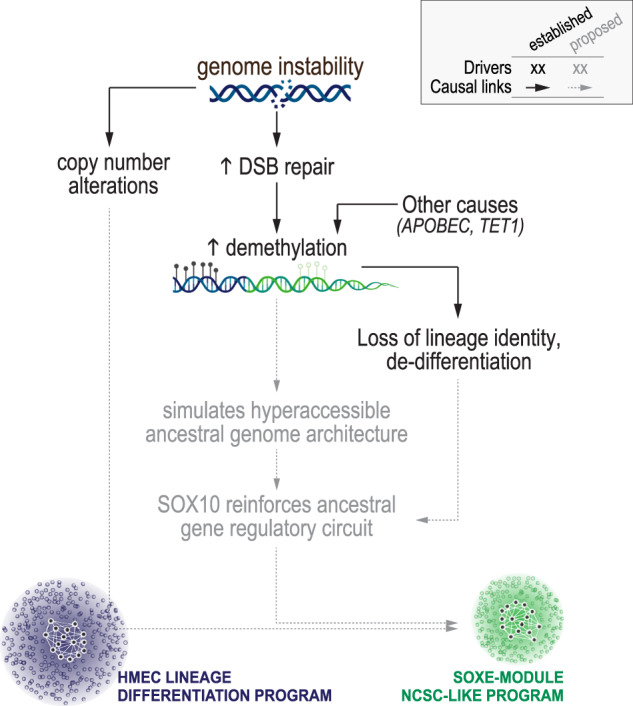
Model summarising the study findings. Proposed links between established drivers of TNBC progression, epigenome erosion and the emergence of a neural crest-like transcriptional programme in de-differentiated TNBCs.

In summary, the SOXE-module’s dominance over the TNBC transcriptome is directly proportional to APOBEC activity, DSB repair and *TET1* expression, which are all demethylating. Of all methylation domains across the genome, the module is most strongly correlated with hypomethylated promoter CGI shores—the substrate for lineage-specific methylation. Kim et al. showed that the minimal genetic requirements for reprogramming postnatal fibroblasts with an NCSC identity are SOX10 expression and the erasure of previous epigenetic memory^34^. We postulate that progressive erosion of the epigenome in SOX10+ tumour-initiating cells simulates these conditions, driving NCSC-like reprogramming and poor clinical outcomes in SOX10 + TNBCs (Fig. 6).

## Discussion

Heterogeneity has emerged as a major bottleneck to effective sub-classification and treatment of cancer, and TNBC is no exception. Post-treatment relapse occurs through clonal expansion of cells with pre-existing, advantageous mutations, but also cell state changes brought about by adaptive epigenetic remodelling—a phenomenon that unites the ‘cancer stem cell’ and ‘epigenetic progenitor’ models of cancer^65^. The intrinsic plasticity of TNBC is problematic because existing therapies cannot eradicate a shifting target. Early evidence implies that blocking this capability with epigenetic therapy may improve treatment efficacy, but this will require a deeper understanding of how phenotypic plasticity evolves^66^. TNBC exhibits genome-wide hypomethylation, which evidently drives de-differentiation by destroying the state-defining epigenetic barcode of its normal cellular precursor, the LP cell^14–17,65,67^. Differential methylation at certain genomic loci is prognostic in TNBC^22^, and myriad studies have helped to decipher the mechanistic contributions of individual writers, readers, and erasers of epigenetic marks, but the phenotypic manifestations of genome-wide 5mC loss have not been extensively studied.

Consistent with functional analysis of Sox10 in experimental mice^29,32^, our human tumour network studies show that *SOX10’s* TNBC-specific regulatory module confers similarity to highly plastic NCSCs. We traced a cluster of super-connected SOXE-module genes back to the tissue-resident mammary stem and progenitor cells and found that in contrast to the normal breast where it was associated with epithelial lineage differentiation, in TNBC this core was connected to Wnt signalling, neuroglial differentiation and embryo patterning genes. Critically, we found that expression of the SOXE-module amongst TNBCs was proportional to overall transcriptional similarity to Sox10+ migratory NCSCs from chick embryos^55^, despite there being minimal direct overlap in member genes. We also identified SOXE-module hub genes as points of maximum network vulnerability as candidate therapeutic targets. In support of this approach, two of these—*BBOX1* and *BCL11A*—have already been validated as such in TNBC^68–72^.

To better understand the evolution of NCSC-like transcriptional reprogramming, we investigated potential links to the established drivers of TNBC development—genomic instability, large-scale CNAs, and defective DNA repair. We identified several processes that correlate significantly with the SOXE-module eigengene (DSB repair, APOBEC and TET1 activity, which are all demethylating); but most discernibly, the loss of lineage-specific methylation marks at CGI shores. Several mechanisms have been postulated to contribute to widespread methylome erosion in cancer, including DSB repair^58,59^ and reduced availability of 5mC substrates through metabolic reprogramming^73^. Accepting that there are probably multiple contributing factors in any individual tumour, our findings nevertheless suggest that NCSC-like reprogramming occurs concomitantly with epithelial de-programming in TNBC. The gene regulatory networks that operate in NCSCs are amongst the most evolutionarily conserved in vertebrates^25,74^. We postulate that when the broadly open chromatin landscape of the early embryo is simulated in epigenetically eroded tumours, dominant fate specifiers like SOX10 may recreate their ancestral regulatory circuits by default.

In summary, our data indicate that the extent of promoter methylation loss in SOX10+ breast tumours correlates with their transcriptomic similarity to NCSCs—the earliest developmental cell state programmed by SOX10 activity and one synonymous with migration, multipotency and phenotypic plasticity. We propose that during TNBC development, progressive erosion of the epigenome drives de-differentiation while simultaneously making cells vulnerable to NCSC-like reprogramming. Broadly, these findings support preclinical data^19–23^ on the potential for epigenetic modulators to combat phenotypic plasticity in TNBC.

## Methods

### Human tissue samples (also see Table 2)

This study involved immuno-detection of SOX10 and other biomarkers in the following human tissue cohorts:

1. Reduction mammoplasty (RM) samples: obtained in collaboration with Dr William Cockburn (Wesley Hospital, Brisbane) and the Royal Brisbane and Women’s Hospital (RBWH) Plastics Unit. Nineteen RM specimens were used for IHC and IF analysis, and two for methylation arrays. Age, parity and menopausal status of these patients were unknown. 30% of cases showed fibrocystic change and 10% presented with columnar cell lesions (histopathology review by SRL).
2. Clinically annotated, primary breast tumour samples:
  - A cross-sectional primary breast tumour cohort comprising samples from Australia (treated by the RBWH Breast Unit) and the UK (Nottingham University Hospital), from patients treated in the mid-1980s to mid-1990s. Tumour blocks were sampled as 0.6 mm cores in tissue microarrays (TMAs). For baseline characteristics see Supplementary Table 2.
  - Metaplastic carcinomas (Asia-Pacific Metaplastic Breast Cancer Consortium (whole sections).
3. Patient-matched primary TNBC and brain metastases (*n* = 19 pairs). Tumour blocks were sampled as 1.0 mm cores in TMAs.

### Ethics approval

Human research ethics approval was obtained from the Royal Brisbane and Women’s Hospital (2005000785), The University of Queensland (HREC/2005/022) and North West Greater Manchester Central Health (15/NW/0685). Written patient consent to use tissue for research purposes was obtained where required under the conditions of these approvals and all samples were de-identified in the analytical database. This study complies with the World Medical Association Declaration of Helsinki.

### Immunohistochemistry (IHC)

Formalin-fixed, paraffin-embedded (FFPE) tissue samples or TMAs were sectioned, deparaffinised, subjected to antigen retrieval and chromogenically stained as described in ref. ^75^ and detailed in Supplementary Table 1. Slides were scanned using the Aperio ScanScope T2 digital scanning system at 40x magnification. TMA images were segmented using Spectrum software (Aperio), and high-resolution images of individual cores were extracted and scored by two experienced observers in a blinded fashion (hidden metadata tags corresponding to TMA position were used to link clinical and sample data). Digital image files were scored according to the criteria set out in the legends to Figs. 2e and S2h.

### Immunofluorescence (IF)

FFPE RM tissue sections (Table 2) were sectioned, deparaffinised, subjected to antigen retrieval and stained as described in ref. ^76^ (Supplementary Table 1). Briefly, primary antibodies diluted in tris-buffered saline (TBS) were incubated on tissue sections for 1 h at room temperature, washed in TBS then incubated with secondary antibodies for 30 min in the dark. To minimise tissue autofluorescence, slides were stained with SUDAN Black for 20 min in the dark (Sigma #S-2380), then washed (0.1% TBS-Tween (30 min), TBS (10 min). Slides were mounted using Vectashield (Vecta Labs) with DAPI (Sigma-Aldrich), cover-slipped, sealed and imaged on a Carl Zeiss MicroImaging system using Axio Vision LE version 4.8.2 (PerkinElmer).

**Table 2:** Biological resources.

Resource	Source, identifier and relevant citations	Related figure(s)
Tissue samples		
Histologically normal breast FFPE whole sections	The Brisbane breast bank^48^	1a–e
Fresh RM surgical samples	The Brisbane breast bank^48,76^	1f, Supp-1b, Supp-6a
Australian BC series, FFPE TMA sections & clinical data	Pathology Qld & The Brisbane breast bank^48,89^	2e, f, 3d–e, Supp-2h-k
UK breast cancer series, FFPE TMA sections & clinical data	Nottingham Breast Cancer Research Centre^90,91^	2e, f, Supp-2h-k
Metaplastic tumour series, FFPE sections & clinical data	Asia-Pacific MBC consortium^51,92^	2g
Patient-matched primary TNBCs and brain metastases	Pathology Qld & The Brisbane breast bank^48,89^	2h
Cancer cell lines		
293 T	ATCC^®^ CRL-3216™	Supp-1a, Supp-2g
MDA-MB-435S	ATCC^®^ HTB-129™	Supp-1a, Supp-2e, Supp-2g
HCC38	ATCC^®^ CRL-2314™	Supp-2e
HCC1569	ATCC^®^ CRL-2330™	Supp-2e, Supp-2g
Primary melanoma cells (D41, D05)	Dr. Chris Schmidt, QIMR Berghofer^77^	Supp-2e
TaqMan gene expression assays		
SOX10	ThermoFisher, Hs00366918_m1	Supp-2e
RPL13A	ThermoFisher, Hs03043885_g1	Supp-2e
shRNA sequences		
SOX10_1	Sigma-Aldrich TRCN0000018984	Supp-1a, Supp-2g
SOX10_2	Sigma-Aldrich TRCN0000018987	Supp-1a, Supp-2g
SOX10_3	Sigma-Aldrich TRCN0000018988	Supp-1a, Supp-2g
Non-targeted negative control (NTNC)	Sigma-Aldrich SHC002	Supp-1a, Supp-2g

*Supp* supplementary.

### Fresh reduction mammoplasty (RM) tissue processing and fluorescence-activated cell sorting (FACS)

RM samples were processed, and single-cell suspensions were prepared as previously described (Table 2 and refs. ^48,76^). Briefly, tissue was cut into small pieces (~5 mm^3^) and digested overnight with agitation at 37 °C in DMEM-F12 (Gibco), foetal bovine serum ((FBS), 5%, Gibco), antibiotic/antimycotic (Gibco), Amphotericin B (2.5 μg/mL, Gibco), collagenase type I-A (200 U/mL, Sigma-Aldrich) and Hyaluronidase I-S (100 U/mL, Sigma-Aldrich). Epithelial organoids were obtained by centrifugation (80 × *g*, 1 min), then dissociated to single-cell suspensions for 5–10 min in TrypLE (Gibco), followed by Dispase (5 mg/mL, Gibco) and DNAse-I (100 ug/mL, Invitrogen). Enzymatic activity was quenched in ice-cold Hank’s Balanced Salt Solution ((HBSS), Gibco) with 2% FBS and cells were filtered through a 40-μm cell strainer (BD Falcon).

Cell concentration and viability were determined using a Countess^®^ automated counter (Invitrogen) with trypan blue and adjusted to 2.0E^6^/mL. Single-cell suspensions (typically 30–60 mL) were labelled for 10 min on ice with Sytox^TM^ green (Invitrogen) plus a cocktail of fluorescent antibody conjugates to discriminate hMEC subsets (negatively gated, non-epithelial ‘lineage’ markers: CD31, CD45, CD140b; positively gated hMEC markers: CD49f, EpCAM—see Supplementary Table 1 and Supplementary Fig 6a). Samples were washed (80×*g*, 2 min) and then resuspended in cold HBSS + 2% FBS. For robust fluorescence compensation and gating of specific hMEC populations, we also tested in parallel small samples stained with isotype control antibodies, and ‘fluorescence minus one’ negative controls (samples from which one of the main conjugates was omitted). Fluorescence data acquisition, gate placement and sorting were performed on a BD FACS Aria II instrument with FACSDiva software (v6.1.3; QIMR Berghofer). Sorted cells were collected on ice before being pelleted (80×*g*, 2 min) and snap-frozen at −70 °C.

### Methylation array profiling and ChIP-seq meta-analysis

DNA was extracted from FACS-sorted hMEC samples using the QIAGEN AllPrep DNA/RNA mini kit, with bisulphite conversion using the EZ DNA methylation Kit (Zymo Research) following the manufacturer’s protocol with modification for Illumina methylation arrays. Bisulphite-converted DNA was amplified and hybridised to Infinium methylationEPIC 850k beadchips (Illumina) according to the manufacturer’s protocol. Arrays were scanned on an iScan, and data were processed using GenomeStudio (Illumina) with BMIQ array normalisation to derive average methylation beta-values.

Histone modification ChIP-seq data were obtained from Pellacani et al.^42^. Bigwig format files were retrieved from www.epigenomes.ca, and the mean signal/bin was plotted across the region chr22:38365030-38396083 for each histone mark in each cell type.

### Analysis of SOX10 expression in cell lines

MDA-MB-435, HCC1569 and HCC38 cells were from the American Type Cell Culture Collection (ATCC; (Table 2); authenticated in our laboratory and cultured according to ATCC recommendations^49^. D41 and D05 melanoma cells were selected from the primary melanoma cell line bank of Dr Chris Schmidt and Prof Nick Hayward (QIMR Berghofer) based on having high and low baseline *SOX10* expression, respectively^77^. Cells were routinely cultured at 37 °C in a humidified atmosphere with 5% CO_2_ and routinely screened for mycoplasma. RNA and protein were extracted from cells in the exponential phase of growth using standard Trizol and RIPA buffer methods^78^. *SOX10* mRNA was quantified relative to *RPL13A* as previously described (ref. ^79^ and Table 2). For Western analysis (MDA-MB-435, HCC1569, HCC38 cells), protein lysates (30 μg) were resolved by SDS-PAGE then SOX10 and β-actin were detected using standard chemiluminescence (Supplementary Table 1).

### Stable-shRNA knockdown of SOX10 in breast cancer cell lines

Three pre-validated *SOX10*-targeted shRNA constructs, and a non-targeting negative control (NTNC) construct (pLKO.1), were purchased from Sigma-Aldrich (Table 2). Plasmid DNA was isolated from overnight bacterial cultures, then lentiviral particles were produced by triple transient transfection of HEK-293T (human embryonic kidney) packaging cells with one of the four transfer plasmids (pLKO.1-puro; 2 μg), together with companion plasmids encoding lentiviral packaging and replication elements (2 μg pHR’8.2ΔR + 0.25 μg pCMV-VSV-G; donated by Dr Wei Shi, QIMR Berghofer). Virus-containing supernatants (in target cell media) were then collected over the following two days and filtered (0.45 μm). MDA-MB-435 target cells were seeded at 3.1 × 10^4^/cm^2^ in six-well plates, then after 24–48 h (at ~50% confluence), cells were infected with filtered viral supernatants, supplemented with 1 mg/mL polybrene (Sigma-Aldrich) for 24 h. Stably transduced cells were then selected with 1 μg/mL puromycin (Sigma-Aldrich) for 2 weeks to eliminate uninfected cells.

### Datasets and processing

TCGA level-3 normalised RNAseq data (*'rnaseqv2 illuminahiseq rnaseqv2 unc edu Level 3 RSEM genes normalised data.data.txt'*) from the Data Analysis Center Firehose (http://firebrowse.org/) were used for all single-gene analyses (Supplementary Figs. 2a, 5e; test group stratification for Supplementary Fig. 3; SOX10 heatstrips in Fig. 3a and Supplementary Fig. 6a, c). Scaled estimate columns of the '*rnaseqv2 illuminahiseq rnaseqv2 unc edu Level 3 RSEM genes data.data.txt'* were used for all other algorithmic analyses.

For methylation datasets, TCGA level-3 Illumina HM450k data were downloaded from the National Cancer Institute Genomics Data Commons (GDC) data portal (https://portal.gdc.cancer.gov/) and processed using the ChAMP package^80^. We applied the champ.filter function to remove problematic probes (those mapping to X/Y chromosomes, mapping to multiple locations, located near an SNP and non-CG probes). Filtered data were normalised using the champ.norm function, according to the Beta-Mixture Quantile (BMIQ) algorithm; is an intra-sample normalisation procedure that corrects the bias of type-2 probe values.

Level-4 GISTIC-2 copy-number data for TCGA cases were downloaded from the Data Analysis Center Firehose (http://firebrowse.org/) and used for correlative analyses with no further processing. To apply tumour purity cutoffs (TCGA cases), we used a consensus measurement of four different purity estimation methods^81^.

With permission from the METABRIC data access committee, normalised Illumina HT 12 expression array data were downloaded from the European Genome-phenome Archive (EGAD00010000210-211). For the ICGC RNAseq dataset, normalised data were downloaded as supplementary data^45^ and used with no further processing. Mutational signature data (COSMIC, v2 SigProfiler) were downloaded as raw event counts from ref. ^45^ and HRDetect probability scores for these cases from ref. ^57^.

### Differential expression analysis of SOX10-high and -low TNBCs (Supplementary Fig. 3)

To characterise the transcriptomic phenotype associated with *SOX10* expression in TNBC, we performed differential expression analysis of *SOX10*-high versus *SOX10*-low (median split) TCGA and METABRIC datasets using limma^82^ (differential expression was defined by a corrected *p* value cutoff of 0.01).

### Ontology enrichment analyses

GO term enrichment analysis was performed using the Generic GO term finder hosted by Princeton University (Lewis-Sigler Institute for Integrative Genomics; https://go.princeton.edu). Gene set enrichment analysis (GSEA) was performed using the Prerank function of GenePattern^83^ using 1000 permutations. For Supplementary Fig 3, GSEA inputs comprised differentially expressed genes (*q* ≤ 0.01) ranked by fold-change in each dataset. The input for all other GSEA experiments was whole transcriptome gene lists ranked by a Spearman correlation coefficient. Biological process genesets (Gene Ontology v7.2; gene set size 15-500) were mined for unsupervised analyses and neural crest genesets for supervised analyses (Supplementary Table 11). Datasets and ranking metrics are indicated in the respective Figure legends. Normalised enrichment scores (NES) and corrected *p* values are reported. GeneGo (Metacore^®^ Clarivate Analytics) and Ingenuity^®^ Pathway Analysis (Ingenuity) were also used to analyse pre-ranked gene lists. REVIGO^84^ was used to resolve semantic redundancy and identify major themes amongst the enriched terms.

### Weighted gene co-expression network analysis (WGCNA)—module identification and validation

WGCNA is a powerful network analysis tool that identifies groups of transcripts (modules) that fluctuate in a highly coordinated fashion, implying co-functionality^52,53^. First, it iteratively correlates the expression of every pair of transcripts in a test dataset, producing an adjacency matrix. It then converts this to a topological overlap matrix that reflects net connection weight, accounting for both direct connections and the impacts of shared neighbours. In this study, we created ‘signed’ networks, which reflect the overall topological overlap considering both positive and negative correlations. Dynamic module identification and characterisation (derivation of network metrics, sample eigengene values and module preservation in orthogonal datasets, see below) were performed in the R coding environment, and publication-quality figures were prepared from raw datasets using GraphPad Prism or Clustergrammer (Table 2).

Modules were identified using the TCGA RNAseq (*n* = 919 samples after quality filtering) and validated using METABRIC (*n* = 1278; expression array; Supplementary Fig 6b–d). A consensus set of eight modules was determined according to satisfactory concordance between these two orthogonal networks and a third was generated from the ICGC dataset (*n* = 342; RNAseq). We further validated the eight consensus modules using preservation analysis on a third breast cancer expression dataset. For normal breast samples, WGCNA was performed independently on TCGA normal breast samples (*n* = 97 after quality filtering).

Standard WGCNA outputs include the following (raw data in Supplementary Tables 5–11):

- Module eigengene (ME): a theoretical gene that is the most strongly connected to all other genes in the module and hence represents net module expression and connectivity. Mathematically, the first principal component of each module’s adjacency matrix.
- Module membership and connectivity: Each gene is ascribed k values describing modular and network connectivity (kTotal, kWithin and kOut). These continuous variables are amenable to integrated analysis of overlapping transcriptional programmes, utilising the granularity in expression datasets rather than levelling it as is done when assigning fixed phenotypes or categories. kME correlation and kME *p* values describe how tightly individual genes are linked to all other genes within each module.
  To identify hub genes (Supplementary Fig. 6e), additional network connectivity and influence measures were calculated for each node in the SOXE-module topological overlap matrix using igraph toolkit functions in R:
- betweenness centrality: betweenness(graph, v = V(graph), directed = FALSE, weights = NULL, nobigint = TRUE, normalised = FALSE).
- eigencentrality: eigencentrality(graph, directed = FALSE, scale = TRUE, weights = NULL, options = arpack defaults).

Finally, we used community detection algorithms^85,86^ to examine the substructure of the SOXE-module (MATLAB 2020a), using the adjacency matrix as input. This revealed a hierarchical, sub-modular organisation, and consistently discriminated two partitions (59 and 41% of nodes each). To identify the module ‘control centre’ and hub genes as points of structural vulnerability, submodule assignment was cross-referenced against clustered Cosine similarity data (Fig. 3b, Clustergrammer^87^) with the same input (Supplementary Fig. 4).

### Neural crest genesets

Geneset-1 (NC terms) comprises 308 genes represented in at least two of the 78 terms matching ‘neural crest’ and ‘human’ in the gene ontology database (http://geneontology.org). Geneset-2 (ch.NCSC) comprises the top 200 transcripts statistically over-represented in Sox10+ chick neural crest cells compared to all other embryo cells (fold-change 3.9–23.3; false discovery rate 9.3E^−03^–1.0E^−15^)^55^ (Supplementary Table 11). The ch.NCSC gene set represents genes coordinately expressed with *Sox10* in a stem cell state hence was also suiTable-for network analyses (see below). We used the *singscore* algorithm^88^ to score RNAseq datasets against the neural crest genesets at the individual sample level.

### Breast cancer methylation data analyses

Methylation beta-values were derived from TCGA level-3 Illumina HM450k data as outlined above. Beta-values for all probes corresponding to TSS1500, TSS200 and 5′UTR regions in each sample were first normalised to correct for their bimodal distribution (median absolute deviation (MAD): *P*_β_ *–* median*(P*_β_ – median(*R*_β_)); where *P* = probe in the promoter region and *R* = all probes in promoter region). After filtering out genes with >2 missing probes and those for which >2% of samples were missing data, the final dataset included average MAD-normalised promoter methylation beta-values for 4482 genes (determined from a total of 518 samples with complete clinical annotation). Pairwise Spearman correlations were then calculated between each promoter region and each module eigengene across the sample cohort. Unsupervised hierarchical clustering of correlation values was performed in R using the *Flashclust* package based on the Euclidean distance method. Clusters were visualised and validated with the *cluster* package, using the Silhouette coefficient to confirm distinct clusters. To generate t-distributed stochastic neighbour embedding (t-SNE) plots, we used the Rtsne package (https://cran.r-project.org/web/packages/Rtsne/) on normalised beta methylation values, with 5000 iterations and a perplexity parameter of 40.

**Table 3:** software, code, and published datasets.

ResRource	Source, identifier and relevant citations	Related figure(s)	Related table(s)
Software packages and code			
ChAMP	https://bioconductor.org/packages/release/bioc/html/ChAMP.html^80^	5d–f	–
Clustergrammer	https://maayanlab.cloud/clustergrammer/^87^	3b	Supp-10
Community detection algorithms	Refs. ^85,86^	Supp-4a	–
Epifactors database	https://epifactors.autosome.ru^61^	Supp-5e	–
FACSDiva™	BD Biosciences, licensed	1f, Supp-6a	–
FCS Express (v7)	De Novo Software, licensed	1f, Supp-6a	–
GSEAPreranked	https://genepattern.org^83^	3c, 4d, 5f, Supp-3	1, Supp-4, Supp-9
Ingenuity Pathways Analysis (IPA)	Ingenuity, licensed	–	1
MATLAB	Mathworks, licensed	Supp-4a	Supp-10
Princeton Generic GO term finder	https://go.princeton.edu^93^	5a	Supp-13, 14
Prism (v8.4.3)	GraphPad, licensed	Multiple	S2
R package, Cluster	https://cran.r-project.org/web/packages/cluster/index.html	5f	–
R package, FlashClust	https://cran.r-project.org/web/packages/flashClust/index.html	5f, g	Supp-14
R package, Limma	https://www.bioconductor.org/packages/release/bioc/html/limma.html	Supp-3	Supp-3
R package, t-SNE	https://CRAN.R-project.org/package=Rtsne	5d	–
R package, WGNCA	https://cran.r-project.org/web/packages/WGCNA/index.html^52,53^	Multiple	Multiple
REVIGO	http://revigo.irb.hr	Supp-3	Supp-4
Singscore	https://www.bioconductor.org/packages/release/bioc/html/singscore.html^88^	4c	–
SPSS	IBM, licensed	–	Supp-2
Tableau desktop (2020.4)	Tableau, licensed	4a	–
Published datasets			
Cell line expression data	https://www.ebi.ac.uk/arrayexpress^47^ (E-TABM-157)	Supp-2e, f	–
Cell line expression, CNA and methylation datasets	https://www.ncbi.nlm.nih.gov/gds^46^ (GSE42944; GSE48216)	Supp-2e, f	–
Chicken embryo neural crest gene set	Ref. ^55^, Supplementary Table 1	4b–d	Supp-11
Gene ontology resource	http://geneontology.org	–	Supp-11
Genomic locations of solo-WCpGW sites	Ref. ^60^	Supp-5c	–
hMEC ChIP-seq data	www.epigenomes.ca; ref. ^42^	1f	–
hMEC gene expression array data	Gene expression omnibus, https://www.ncbi.nlm.nih.gov/geo/ (GSE16997); and ref. ^15^ (Tables S5–8)	1e	–
Human reference genome NCBI build 37 (GRCh37/hg19)	UCSC Genome Browser https://genome.ucsc.edu	2d, Supp-5a	–
ICGC gene expression data	Ref. ^45^, Supplementary Table 7	–	Supp-8
ICGC HRDetect scores	Ref. ^57^, Supplementary Table 3b	5c	–
ICGC mutational signatures (COSMIC, v2 SigProfiler)	Ref. ^45^, Supplementary Table 21B, S21E	5c	–
Illumina Infinium Omni2.5 array data	https://www.ncbi.nlm.nih.gov/geo/ (GSE199579)	1f, Supp-5b	–
METABRIC gene expression & clinical data	EGAD00010000210, EGAD00010000211, EGAS00000000083; EGA portal, via data access committee^43^	2a, 3f, g, Supp-3, Supp-4c, d	Supp-4, Supp-7
MetaCore	https://portal.genego.com	Supp-3	Supp-4
SOXE-module network metrics	This paper	4a, f, 5b, g	Supp-10
TCGA clinicopathologic annotation	Ref. ^94^	2a–d, 3a	–
TCGA gene copy-number data	Gistic2.Level_4; TCGA Data Analysis Center Firehose^44^ https://gdac.broadinstitute.org	2b, 5a, b, Supp-5a	–
TCGA gene-level methylation data	Preprocess/meth.by_min_expr_corr; TCGA Data Analysis Center Firehose^44^ https://gdac.broadinstitute.org	2b, c	–
TCGA Illumina HiSeq RNASeq-v2 RSEM level-3 normalised datasets	illuminahiseq_rnaseqv2-RSEM_genes_normalized (MD5); TCGA Data Analysis Center Firehose^44^ https://gdac.broadinstitute.org	2a, c	Supp-4
TCGA Illumina HiSeq RNASeq-v2 RSEM level-3 raw counts	TCGA Data Analysis Center Firehose^44^ https://gdac.broadinstitute.org	3a, S3	Supp-3, 5, 6, 9, 10, 12, 13
TCGA probe-level methylation data	Humanmethylation_450; TCGA Data Analysis Center Firehose^44^ https://gdac.broadinstitute.org	5d–f, Supp-5b–d	–
Triple-negative breast cancer subtypes (Burstein et al)	Ref. ^39^, Supplementary Table 19	3f, Supp-2b	–
Tumour purity for TCGA cases	Supp data-1 (CPE metric) & infinium metric, refs. ^81,95^	Multiple	–
WGCNA ME dataset, ICGC cases	This paper	Multiple	Supp-8
WGCNA ME dataset, METABRIC cases	This paper	Multiple	Supp-7
WGCNA ME dataset, TCGA normal cases	This paper	Multiple	Supp-12
WGCNA ME dataset, TCGA tumour cases	This paper	Multiple	Supp-6
WGCNA mod membership dataset (TCGA cohort)	This paper	Multiple	Supp-5

*Supp* supplementary.

### Reporting summary

Further information on research design is available in the Nature Research Reporting Summary linked to this article.

## Supplementary information

Supplementary data tables

Supplementary figures

Reporting Summary Checklist

## Data Availability

Published datasets used in this paper are outlined in Table 3. Network data generated by the study are also outlined in Table 3, and available as supplementary data. Raw DNA methylation array data for FACS-sorted normal breast epithelial cell subsets are available from the Gene Expression Omnibus (GSE199579; Table 3).

## Code availability

This study used published code and/or publicly available tools (see Table 3).
