# Supplementary material for: Epigenome erosion and SOX10 drive neural crest phenotypic mimicry in triple-negative breast cancer
Source: NPJ Breast Cancer. 2022 May 2;8:57. doi: 10.1038/s41523-022-00425-x (PMC9061835; doi:10.1038/s41523-022-00425-x)
Supplement: Supplementary file 2 — Supplementary figures [file 41523_2022_425_MOESM2_ESM.pdf]

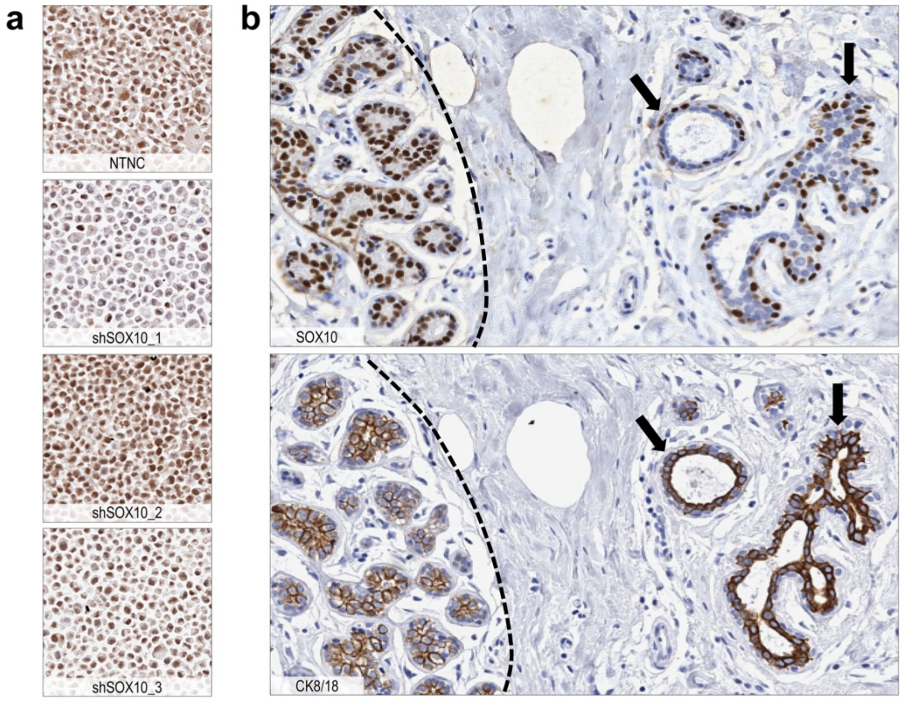

Supplementary Figure-1: Data supporting Figure-1.

(**a**) Validation of SOX10 antibody specificity in FFPE MDA-MB-435 cell pellet sections. Prior to fixation and analysis, cells were stably transduced with non-template negative control (NTNC) or *SOX10* shRNAs. (**b**) IHC analysis of SOX10 and cytokeratin (CK) 8/18 expression in serial RM tissue sections. Representative image shows a lobule with SOX10+ luminal epithelia and weak expression of CK8/18 (outlined); and nearby ducts (arrows) in which the luminal epithelium lacks SOX10 expression but stains strongly for CK8/18.

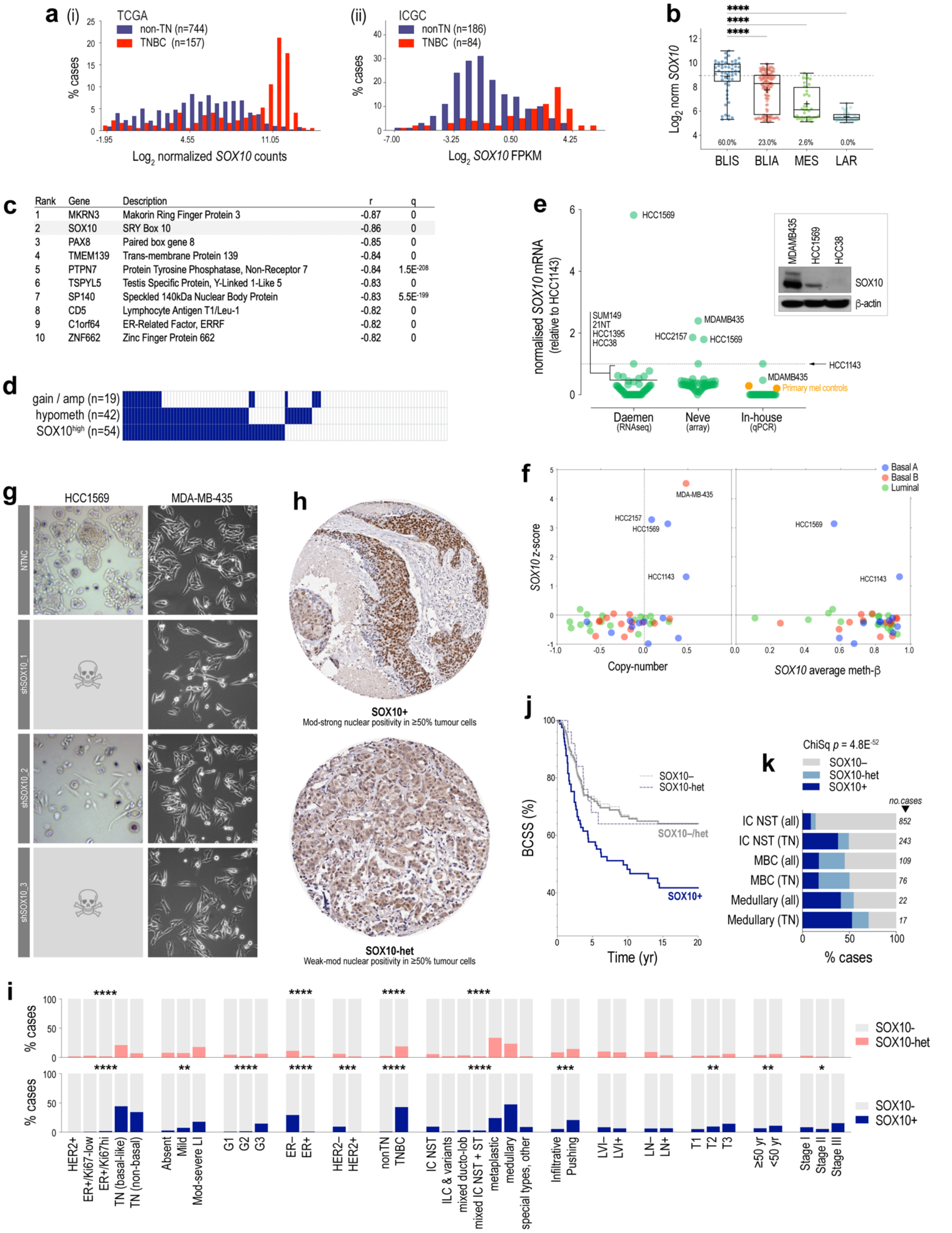

Supplementary Figure-2: Data supporting Figure-2.

(**a**) *SOX10* mRNA distribution in TNBC and non-TN cases from TCGA and ICGC cohorts. (**b**) Expression of SOX10 mRNA in the METABRIC dataset according to TNBC subtypes defined by Burstein et al. BLIS, basal-like immune-suppressed; BLIA, basal-like immune-activated; MES, mesenchymal; LAR, luminal androgen receptor-like. Dotted line, 75th percentile for all samples. (ANOVA test: ****p<0.0001). (**c**) Of all genes expressed in breast cancer, *SOX10* has the second strongest relationship with gene methylation (Broad Institute TCGA Genome Data Analysis Centre). (**d**) Percentages of *SOX10* hypomethylated (90th percentile of melanoma values) and copy-number altered

cases (GISTIC). *amp, amplified*. (**e**) Expression of *SOX10* mRNA in breast cancer cell lines; three independent datasets shown. Inset: protein expression in selected lines determined by Western analysis (antibody validation in Supplementary Figure-1). (**f**) Relationships between *SOX10* expression, copy-number and gene-averaged methylation beta values in breast cancer cell lines. The high levels of promoter methylation and barely detectable mRNA in the majority of lines suggested that *SOX10* silencing is a cause and/or consequence of *in vitro* adherent selection. Culturing a range of lines as non-adherent tumourspheres was not sufficient to reactivate SOX10 expression (data not shown). (**g**) Light microscopy images (20x magnification) comparing cell morphology and cell death (☠) of MDA-MB-435 melanoma and HCC1569 basal-like breast cancer cell lines stably transduced with *SOX10*-targeted shRNA, or a non-targeted negative control (NTNC) hairpin. HCC1569-sh*SOX10* derivatives did not survive beyond three passages after antibiotic selection. MDA-MB-435 were viable over multiple (>10) passages but assumed more mesenchymal morphology. (**h**) Additional IHC images showing strong positivity, heterogeneous staining and tumour-associated normal staining in TNBC samples. The heterogeneous core also exemplifies cytoplasmic staining, which was detected in both ER+ and ER– cases and was not associated with any of the clinico-pathologic variables (Supplementary Table-2) that we analysed (data not shown). (**i**) Proportions of SOX10-neg versus SOX10-heterogeneous (upper panel) and positive (lower panel). Chi Square test p-values are shown: *$p<0.01$; **$p<0.001$; ***$p<0.0001$; ****$p<0.00001$. (**j**) Overlapping survival curves for SOX10-neg and heterogeneous staining in TNBC. (**k**) SOX10 heterogeneity in metaplastic and medullary TNBCs compared to tumours of no special type (NST).

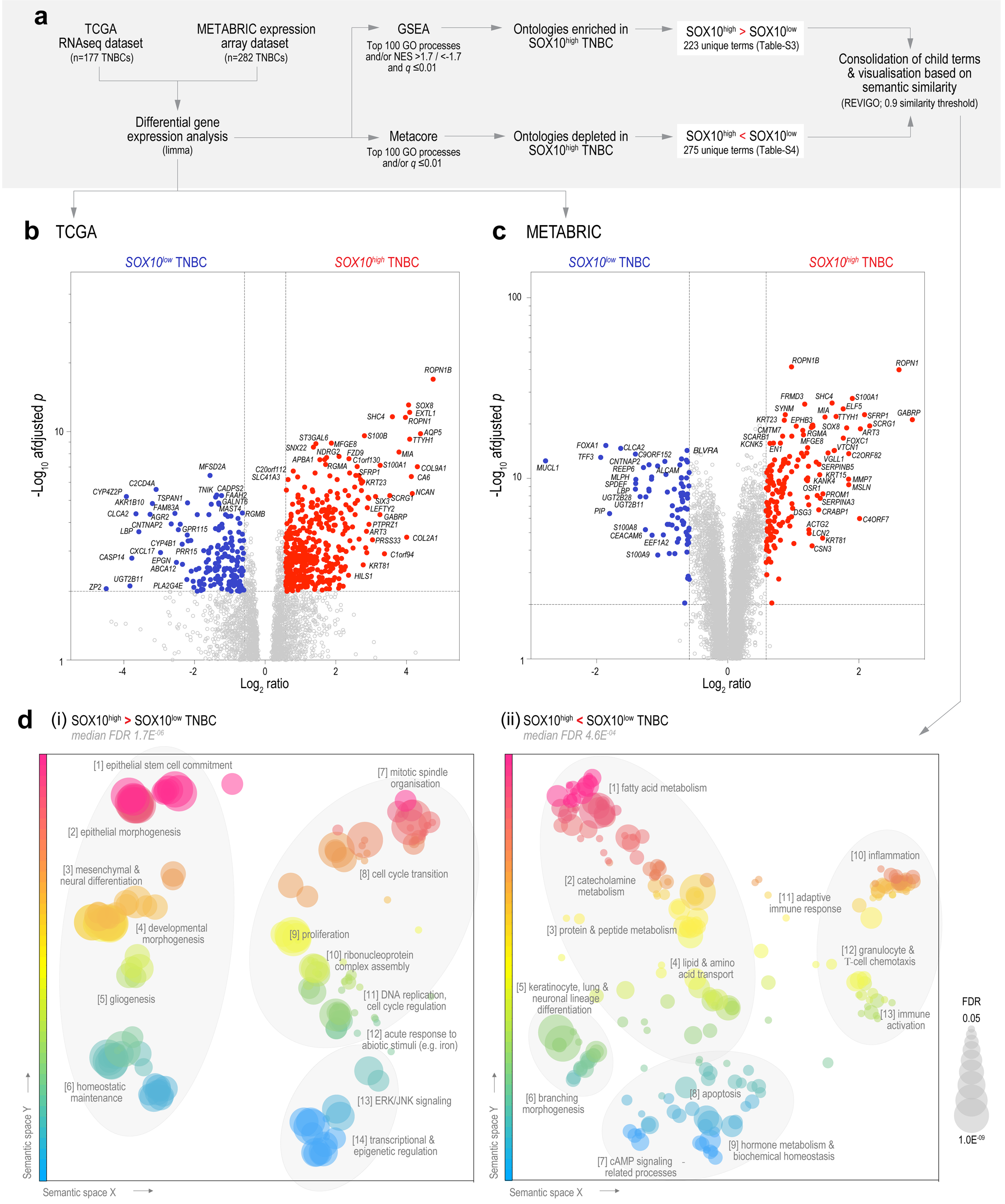

Supplementary Figure-3: Gene ontology profiles of SOX10-high and –low TNBCs.
(**a**) TNBC expression analysis strategy. (**b/c**) Volcano plots indicate transcripts with high significance in differential expression analysis (also see Supplementary Table-3). (**d**) Semantic similarity plots summarising major gene ontologies (GO) enriched in *SOX10*-high and -low TNBCs. Differential gene expression analysis was performed separately on TCGA and METABRIC TNBC datasets. Up to 100 of the most significant (corrected *p*≤0.05) GO processes identified through enrichment analysis (MetaCore and GSEA) were clustered in 2D semantic space using REVIGO ('reduce & visualise gene ontology'), which removes redundancy and assigns X-Y coordinates on the basis of semantic similarity analysis of pre-computed information content[1]. GO terms are coloured according to Y coordinates and sized proportionally to the corrected enrichment p-value (FDR, false discovery rate).

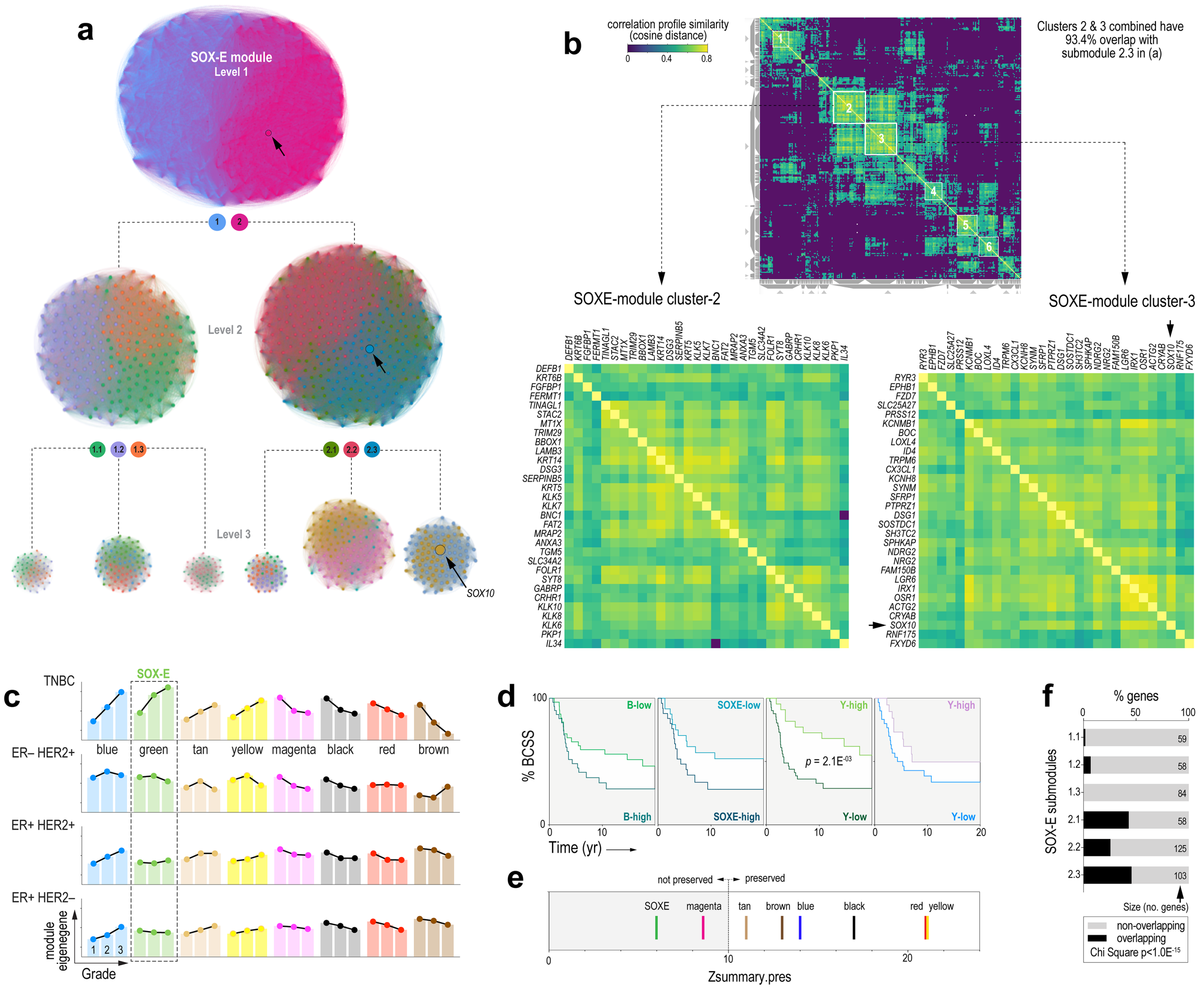

Supplementary Figure-4: Data supporting Figure-3. (**a/b**) Identification of SOXE-module hubs using community detection algorithms to analyse topological structure (a) and unsupervised clustering to map gene correlation profile similarity (according to cosine distance) (TCGA datasets). According to these complementary approaches, approximately 60 genes are most essential to SOXE-module architecture and information flow (89% of submodule 2.3 genes from (a) cluster together in (b). Conversely, 93.4% of clusters 1+2 genes from (b) are located within submodule 2.3 from (a). See also Supplementary Table-12. (**c**) Relationship between SOXE-module expression and histological grade in breast cancer (METABRIC dataset). (**d**) Kaplan Meier analysis of the effects of module co-expression in TNBCs from patients treated with chemotherapy and/or radiotherapy (METABRIC dataset; treatment subpopulation from the cohort in Figure-3g). BCSS, breast cancer-specific survival. ME fraction thresholds for classifying cases as high or low were 0.33 for SOXE/blue and 0.1 for yellow. (**e**) Breast cancer module preservation in normal breast samples (n=50, TCGA). The z-score threshold considered to represent preservation is 10 (indicated). (**f**) Proportions of SOXE-submodule genes shared with *SOX10's* normal breast module, showing significant enrichment in submodules 2.1 and 2.3 in particular

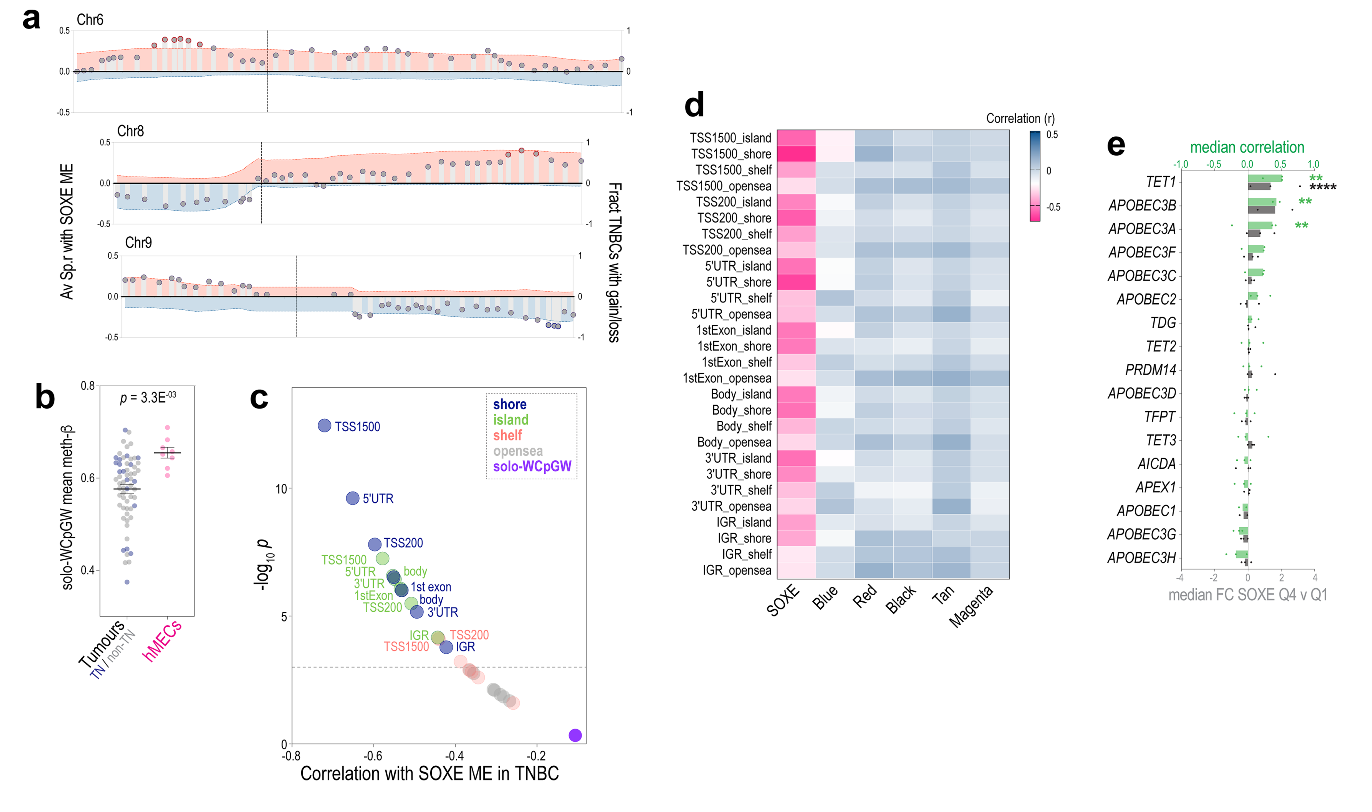

Supplementary Figure-5: Data supporting Figure-5.

(**a**) Relationship between SOXE-ME values and large-scale copy-number alterations (CNAs) on chromosomes 6, 8 and 9 (TCGA datasets). X-axis, chromosome position, to scale; left y-axis and bars: spearman correlation between SOXE-ME values and genes, averaged across cytobands; right y-axis and shaded area: average copy-number gain/amplification (red) and loss/deletion (blue) for genes averaged across cytobands; dotted lines, centromere position. Red/blue circles highlight loci with the highest and lowest correlation coefficients, which generally coincide with TNBC's most frequently gained/lost regions, respectively. (**b**) Average Illumina EPIC 850k methylation array beta values for solo-WCpGW sites in breast tumours versus FACS-sorted hMEC subtypes (see ref[2] and Figure-1). Stats: Mann-Whitney test. (**c**) Correlation (x-axis) and significance (y) of methylation beta values (averaged for the categories listed) versus SOXE-ME values in TNBC. (**d**) Complete correlation matrix for all methylation categories and all WGCNA modules expressed in TNBC (TCGA dataset). (**e**) Relationships between expression of the SOXE module and demethylases in the EpiFactors database[3]. Plots summarise average Spearman correlations between the expression of demethylating enzymes and SOXE ME values (y-axis), and average fold-change (FC; Mann-Whitney test) between TNBCs expressing high (quartile-4) and low (quartile-1) levels of the demethylases (n=3 expression datasets: TCGA, METABRIC, ICGC). **p<0.01; ****p<0.0001.

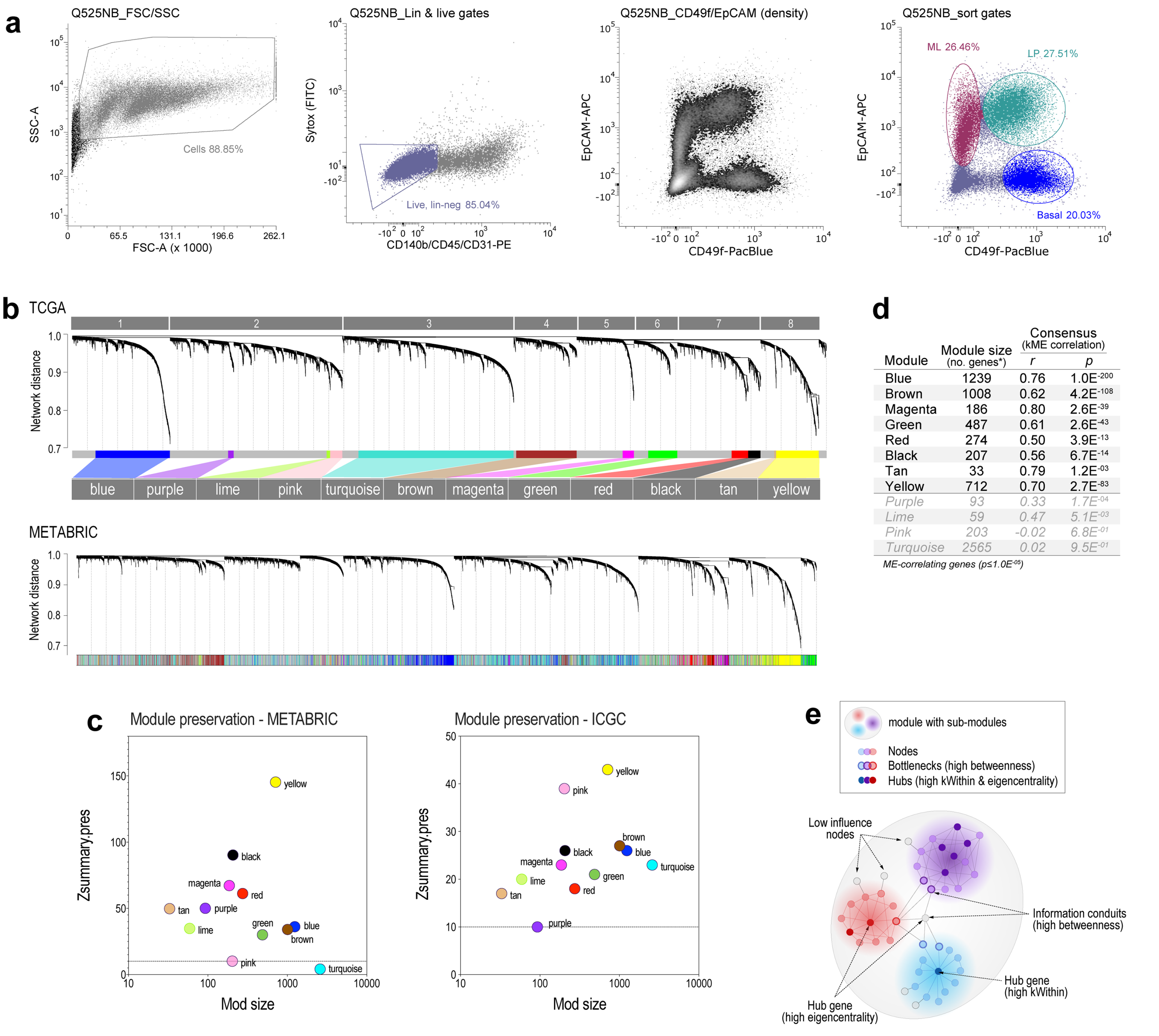

Module	Module size (no. genes*)	Consensus (kME correlation) r	p
Blue	1239	0.76	$1.0E^{-200}$
Brown	1008	0.62	$4.2E^{-108}$
Magenta	186	0.80	$2.6E^{-39}$
Green	487	0.61	$2.6E^{-43}$
Red	274	0.50	$3.9E^{-13}$
Black	207	0.56	$6.7E^{-14}$
Tan	33	0.79	$1.2E^{-03}$
Yellow	712	0.70	$2.7E^{-83}$
Purple	*93*	*0.33*	$1.7E^{-04}$
Lime	*59*	*0.47*	$5.1E^{-03}$
Pink	*203*	*-0.02*	$6.8E^{-01}$
Turquoise	*2565*	*0.02*	$9.5E^{-01}$

ME-correlating genes ($p \leq 1.0E^{-05}$)

Supplementary Figure-6: Methods figures.

(**a**) Representative cytometry plots for sample "Q525NB" demonstrating the gating strategy used for FACS sorting of reduction mammoplasty samples. After gating whole cells from debris based on white light forward and side-scatter (FSC/SSC), negative gating of non-epithelial lineages was performed with a PE primary antibody conjugate cocktail (CD140b+CD45+CD31), then epithelial cell subtypes were discriminated based on CD49f/EpCAM profile as indicated. Percentages of parent gates are shown (whole cells>live/lineage-neg). See Supplementary Table-1 for antibody details and staining conditions. Mature luminal (ML), luminal progenitor (LP) and basal cells were sorted from single-cell suspensions of two tissue donors using this strategy, then analysed using Illumina EPIC 850k methylation arrays to study differential methylation at the SOX10 locus on chromosome 22 (Figure-1f, top panel). (**b**) Breast cancer WGCNA network topological overlap visualized after clustering, where lower values on the y-axis indicate progressively shorter inter-gene distance. The TCGA dataset was used for discovery, and METABRIC for validation. After METABRIC module identification, genes were given the same module assignment to highlight overlap in clustering amongst those with the lowest distance. (**c**) Module preservation. Plots: z-score measures of comparison distribution (>10 considered concordant). (**d**) Module membership correlation between TCGA and METABRIC datasets. The bottom four modules were filtered out based on low correlation between datasets. (**e**) Network schematic defining nodes, hubs, modules, and node influence metrics.
