# Supplementary material for: Epigenome erosion and SOX10 drive neural crest phenotypic mimicry in triple-negative breast cancer
Source: NPJ Breast Cancer. 2022 May 2;8:57. doi: 10.1038/s41523-022-00425-x (PMC9061835; doi:10.1038/s41523-022-00425-x)
Supplement: Supplementary file 3 — Reporting Summary Checklist [file 41523_2022_425_MOESM3_ESM.pdf]

nature portfolio

Corresponding author(s): Jodi Saunus
Sunil Lakhani

Last updated by author(s): 2022/01/13

Reporting Summary

Nature Portfolio wishes to improve the reproducibility of the work that we publish. This form provides structure for consistency and transparency in reporting. For further information on Nature Portfolio policies, see our Editorial Policies and the Editorial Policy Checklist.

Statistics

For all statistical analyses, confirm that the following items are present in the figure legend, table legend, main text, or Methods section.

n/a	Confirmed	
☐	☒	The exact sample size (*n*) for each experimental group/condition, given as a discrete number and unit of measurement
☒	☐	A statement on whether measurements were taken from distinct samples or whether the same sample was measured repeatedly
☐	☒	The statistical test(s) used AND whether they are one- or two-sided<br>*Only common tests should be described solely by name; describe more complex techniques in the Methods section.*
☐	☒	A description of all covariates tested
☐	☒	A description of any assumptions or corrections, such as tests of normality and adjustment for multiple comparisons
☐	☒	A full description of the statistical parameters including central tendency (e.g. means) or other basic estimates (e.g. regression coefficient) AND variation (e.g. standard deviation) or associated estimates of uncertainty (e.g. confidence intervals)
☐	☒	For null hypothesis testing, the test statistic (e.g. *F*, *t*, *r*) with confidence intervals, effect sizes, degrees of freedom and *P* value noted<br>*Give P values as exact values whenever suitable.*
☒	☐	For Bayesian analysis, information on the choice of priors and Markov chain Monte Carlo settings
☒	☐	For hierarchical and complex designs, identification of the appropriate level for tests and full reporting of outcomes
☐	☒	Estimates of effect sizes (e.g. Cohen's *d*, Pearson's *r*), indicating how they were calculated

Our web collection on statistics for biologists contains articles on many of the points above.

Software and code

Policy information about availability of computer code

Data collection	The R package ChAMP was used for raw methylation data processing
Data analysis	This study used a range of published datasets, software packages and tools, which are disclosed in full in the data availability statement and Table-3.

For manuscripts utilizing custom algorithms or software that are central to the research but not yet described in published literature, software must be made available to editors and reviewers. We strongly encourage code deposition in a community repository (e.g. GitHub). See the Nature Portfolio guidelines for submitting code & software for further information.

Data

Policy information about availability of data

All manuscripts must include a data availability statement. This statement should provide the following information, where applicable:

- Accession codes, unique identifiers, or web links for publicly available datasets
- A description of any restrictions on data availability
- For clinical datasets or third party data, please ensure that the statement adheres to our policy

Module membership information for individual genes, and module eigengene values for TCGA, METABRIC and ICGC samples, are reported in supplementary tables. Illumina Infinium Omni2.5 array data for sorted normal breast cell samples is available through the EGA data access process (see Table-3)

Field-specific reporting

Please select the one below that is the best fit for your research. If you are not sure, read the appropriate sections before making your selection.

☒ Life sciences ☐ Behavioural & social sciences ☐ Ecological, evolutionary & environmental sciences

For a reference copy of the document with all sections, see nature.com/documents/nr-reporting-summary-flat.pdf

Life sciences study design

All studies must disclose on these points even when the disclosure is negative.

Sample size	Sample size was not predetermined in this study. For IHC analyses, TNBC cohort sizes were similar or larger than peer-reviewed biomarker studies with a similar design. For computational studies,
Data exclusions	Cases/samples were only excluded if correlative data required to complete the analysis was missing.
Replication	IHC and computational findings were verified in independent cohorts (with orthogonal platforms/methods where applicable).
Randomization	The analyses in this paper were observational.
Blinding	Investigators who performed IHC scoring and computational analyses were blinded to sample and clinical information.

Reporting for specific materials, systems and methods

We require information from authors about some types of materials, experimental systems and methods used in many studies. Here, indicate whether each material, system or method listed is relevant to your study. If you are not sure if a list item applies to your research, read the appropriate section before selecting a response.

Materials & experimental systems

n/a	Involved in the study	
☐	☒	Antibodies
☐	☒	Eukaryotic cell lines
☒	☐	Palaeontology and archaeology
☒	☐	Animals and other organisms
☒	☐	Human research participants
☒	☐	Clinical data
☒	☐	Dual use research of concern

Methods

n/a	Involved in the study	
☒	☐	ChIP-seq
☐	☒	Flow cytometry
☒	☐	MRI-based neuroimaging

Antibodies

Antibodies used	Table-S1 provides a comprehensive description of the 26 antibodies used in this study.
Validation	Antibodies were selected based on use in clinical diagnostic practice (e.g., ER, PR, HER2, Ki67), or in well-cited, peer-reviewed publications. Since SOX10 was central to the paper and our conclusions, we validated this antibody via IHC analysis of FFPE cell pellets, made after stable transduction with SOX10-specific shRNAs (Fig-S1).

Eukaryotic cell lines

Policy information about cell lines

Cell line source(s)	ATCC
Authentication	Cell lines were authenticated by STR profiling.
Mycoplasma contamination	All cell lines were routinely checked for mycoplasma and confirmed to be negative.
Commonly misidentified lines (See ICLAC register)	MDA-MB-435 cells have been the subject of controversy over whether they are of breast or melanoma origin. The most compelling data suggest the latter (e.g., Lacroix 2009, Hollestelle 2009, Cancer Research), however this is not particularly relevant to our study since we used the line purely for its SOX10+ status, to validate an antibodies and shRNA sequences.

Flow Cytometry

Plots

Confirm that:

☒ The axis labels state the marker and fluorochrome used (e.g. CD4-FITC).

☒ The axis scales are clearly visible. Include numbers along axes only for bottom left plot of group (a 'group' is an analysis of identical markers).

☒ All plots are contour plots with outliers or pseudocolor plots.

☒ A numerical value for number of cells or percentage (with statistics) is provided.

Methodology

Sample preparation	Preparation of single cell suspensions from reduction mammoplasty samples is detailed in the methods section.
Instrument	BD FACS Aria II
Software	FACS Diva software (BD, v6.1.3)
Cell population abundance	Sorted sample purity of >99% was confirmed by re-analysing a small aliquot of sorted cells within the gating framework of each sort.
Gating strategy	Debris and doublets/clumps were first gated out of the prepared suspensions based on FSC/SSC characteristics and Sytox-blue positivity. Non-epithelial cells were gated out based on positivity for 'lineage' markers: CD31, CD45 or CD140b. Epithelial subsets were then defined on a CD49f/EpCAM quadrant plot as follows: mature luminal cells (EpCAM+/CD49f-), LP cells (CD49f+/EpCAM+), basal cells (CD49f+/EpCAM-) and undefined (CD49f-/EpCAM-). Gates were placed based on the fluorescence of samples stained with isotype control antibodies (see Table-S1).

☒ Tick this box to confirm that a figure exemplifying the gating strategy is provided in the Supplementary Information.
